# Polyploid giant cancer cells are dependent on cholesterol for progeny formation through amitotic division

**DOI:** 10.1038/s41598-022-12705-4

**Published:** 2022-05-27

**Authors:** Shai White-Gilbertson, Ping Lu, Ikechukwu Esobi, Jing Echesabal-Chen, Patrick J. Mulholland, Monika Gooz, Besim Ogretmen, Alexis Stamatikos, Christina Voelkel-Johnson

**Affiliations:** 1 Department of Microbiology and Immunology, Medical University of South Carolina, Basic Science Building, MSC250504, 173 Ashley Ave., Charleston, SC USA; 2 Department of Food, Nutrition, and Packaging Sciences, Clemson University, Clemson, USA; 3 Department of Neuroscience, Medical University of South Carolina, Charleston Alcohol Research Center, Charleston, USA; 4 Department of Drug Discovery and Biomedical Sciences, Medical University of South Carolina, Charleston, USA; 5 Department of Biochemistry and Molecular Biology, Medical University of South Carolina, Charleston, USA

**Keywords:** Cancer, Cell biology

## Abstract

Polyploid Giant Cancer Cells (PGCC) are increasingly being recognized as drivers of cancer recurrence. Therapy stress promotes the formation of these cells, which upon stress cessation often successfully generate more aggressive progeny that repopulate the tumor. Therefore, identification of potential PGCC vulnerabilities is key to preventing therapy failure. We have previously demonstrated that PGCC progeny formation depends on the lysosomal enzyme acid ceramidase (ASAH1). In this study, we compared transcriptomes of parental cancer cells and PGCC in the absence or presence of the ASAH1 inhibitor LCL521. Results show that PGCC express less INSIG1, which downregulates cholesterol metabolism and that inhibition of ASAH1 increased HMGCR which is the rate limiting enzyme in cholesterol synthesis. Confocal microscopy revealed that ceramide and cholesterol do not colocalize. Treatment with LCL521 or simvastatin to inhibit ASAH1 or HMGCR, respectively, resulted in accumulation of ceramide at the cell surface of PGCC and prevented PGCC progeny formation. Our results suggest that similarly to inhibition of ASAH1, disruption of cholesterol signaling is a potential strategy to interfere with PGCC progeny formation.

## Introduction

The plasticity and adaptability of cancer cells are major therapeutic challenges. Recently, more attention has been focused on a particular driver of this plasticity, a process whereby cancer cells de-differentiate and resume replication from a genetically unstable position, resulting in increased heterogeneity^1–3^. This adaptive process is triggered by stress like hypoxia or treatments like chemotherapy or radiation^4–6^. In response cancer cells cease mitosis and, depending on inherent mutations of genes involved in cell cycle regulation, undergo endoreplication, which results in acquisition of multiple genomes in parallel with de-differentiation that reactivates embryonic programs^7,8^. The resulting polyploid giant cancer cells (PGCC) may hold dozens of genome copies, which are then parceled out to progeny that are created through primitive cell division like budding or bursting^9,10^. PGCC have been suggested to facilitate cancer recurrence after initial therapy response or apparent remission through progeny formation^2,11,12^. Due to the genetic instability of PGCC, progeny that resume mitosis are heterogeneous and include clones that successfully evolve with more aggressive characteristics. Thus, preventing PGCC from successfully generating progeny is an urgent clinical matter.

We previously established that mitotic proliferation in cancer cells occurs independent of the lysosomal enzyme acid ceramidase (ASAH1) but that PGCC progeny generation by primitive cell division is highly dependent on the activity of this enzyme^13^. In order to understand why PGCC but not mitotically proliferating cells depend on ASAH1 function for survival, we performed an unbiased transcriptomic analysis using bulk RNA-seq. We found that more than 8000 genes were differentially expressed between parental cells and their PGCC derivatives whereas inhibition of ASAH1 did not change the expression of any genes in parental cells and only increased the expression of four transcripts in PGCC, including two involved in cholesterol synthesis. In this study, we show that PGCC are highly dependent on cholesterol and that accumulation of ceramide appears to displace cholesterol at the cell surface. The competition between cholesterol and ceramide at the plasma membrane may impact membrane fluidity and the ability of PGCC to generate progeny by primitive cleavage.

## Results

### Analysis of transcriptomes between parental cancer cells and PGCC generated after radiation stress reveal major changes in metabolic pathways involved in protein and DNA synthesis

**Figure 1:**
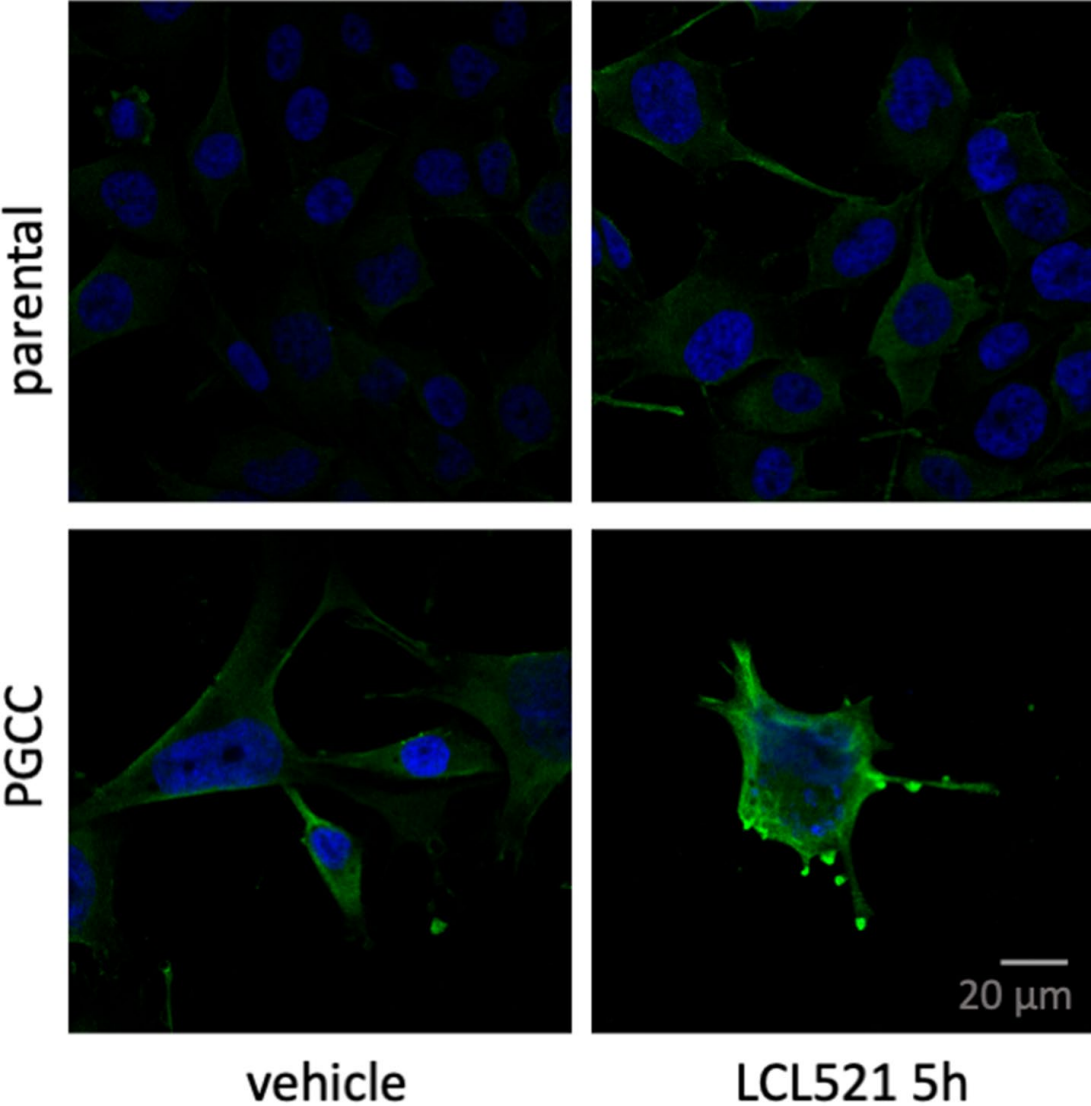
Ceramide accumulation after 5 h of LCL521 treatment. Parental and PGCC cells were treated with vehicle or 5 μM LCL521 for 5 h and ceramide staining analyzed by confocal microscopy. The green signal indicates ceramide staining. Nuclei are identified by DAPI (blue).

**Figure 2:**
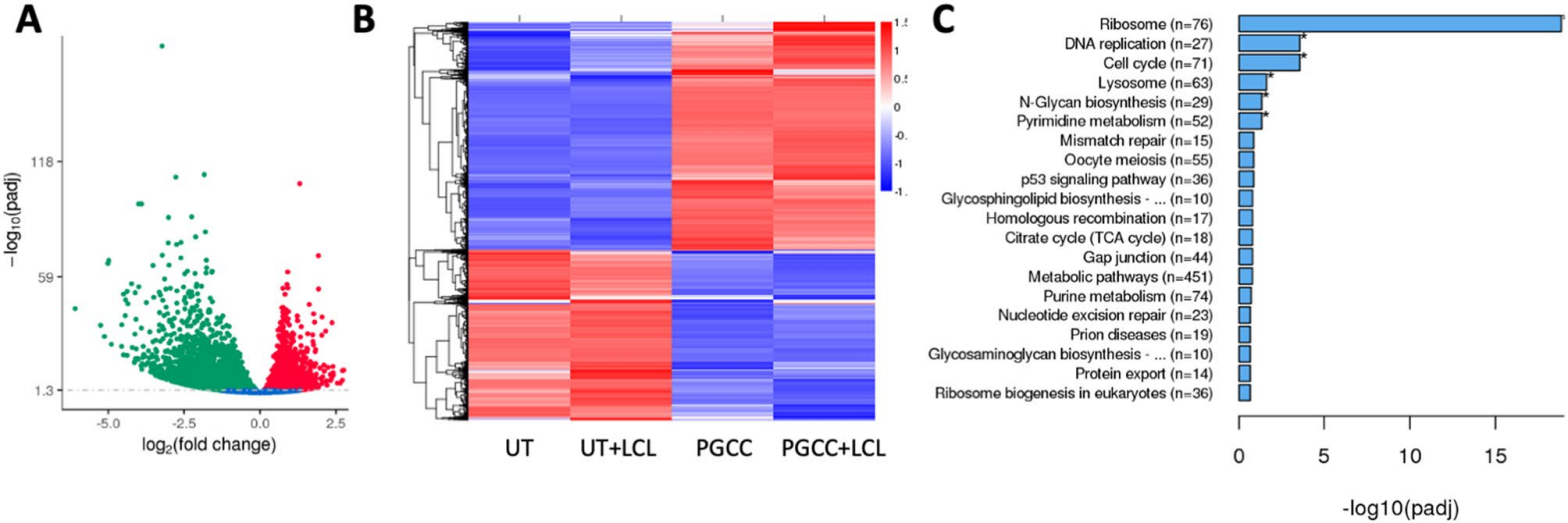
Transcriptomics-identified differences between parental PPC1 cells and PGCC generated through radiation. (**A**) Volcano plot and (**B**) heat map of differentially expressed genes between untreated parental cells (UT) and PGCC (**C**) Top 20 KEGG bioinformatics groups altered in PGCC. Analysis was performed by Novogene using the DESeq2 R package (v.2_1.6.3).

We previously established that inhibition of acid ceramidase (ASAH1) with LCL521 results in a preferential increase in long chain ceramides within 5 h as quantified by LC/MS^13^. To visualize the increase in ceramide, we performed confocal microscopy of parental PPC1 prostate cancer cells and PGCC at 5 h after treatment with 5 μM LCL521. As shown in Fig. 1, LCL521 treatment preferentially increased ceramide at the cell surface of PGCC. Next, we analyzed transcriptomes under the same conditions to determine if a 5-h exposure to LCL521 is sufficient to impact transcriptional programs that could provide insight into PGCC biology. Analysis of transcriptomes between untreated cells and PGCC enriched by filtration revealed significant differences, whereas LCL521 treatment had minimal effects (Fig. 2a). A total of 8125 genes were differentially expressed between parental cells and their PGCC derivatives with 3552 genes upregulated and 4573 genes downregulated in PGCC compared to untreated parental cells (Fig. 2b). KEGG analysis indicated that major differences occur in genes pertaining to ribosomes, DNA replication, cell cycle, the lysosome, N-Glycan biosynthesis and pyrimidine metabolism (Fig. 2c and Fig. S1-S6). Ribosomal genes were decreased whereas genes involved in DNA synthesis were increased, suggesting that PGCC repress protein synthesis in favor of DNA synthesis. Since PGCC are not proliferating, significant differences are also detected in cell cycle genes.

### Inhibition of ASAH1 does not affect gene expression in parental cells but increases select genes in PGCC

**Figure 3:**
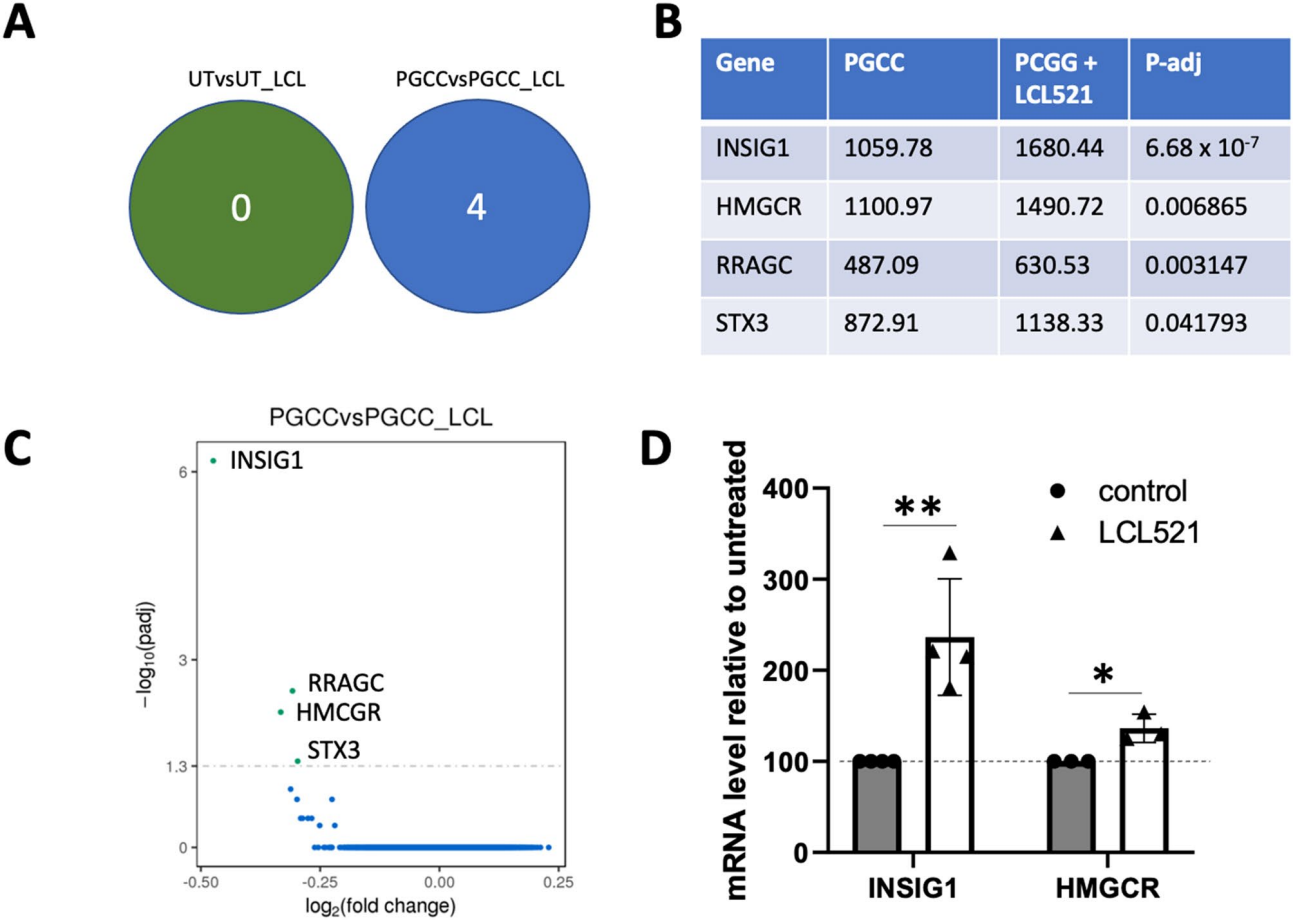
Transcriptomics-identified differences between untreated PGCC and PGCC treated with LCL521 for 5 h. (**A**) Four mRNAs were increased by LCL521 exposure in the PGCC population while none were altered by the drug exposure in the parental PPC1 population. (**B**) Summary of the four upregulated genes in PGCC; all genes were upregulated upon LCL521 treatment. Values in PGCC and PGCC treated with LCL521 as well as the adjusted p-value are shown with p values adjusted using the Benjamini and Hochberg approach for controlling the false discovery rate. (**C**) Graphical representation of change magnitude for the four significantly upregulated mRNAs in PGCC. (**D**) qPCR analysis of HMCGR and INSIG1 in MEL624 PGCC generated through radiation and left untreated or exposed to 5 μM LCL521 for 5 h.

Exposure to 5 μM LCL521 for 5 h did not significantly change the expression of any genes in untreated parental PPC1 cells but significantly upregulated the expression of four genes in PGCC including two, HMGCR (3-Hydroxy-3-Methylglutaryl-Coenzyme A Reductase) and INSIG1 (Insulin Induced Gene 1), that are involved in the synthesis of cholesterol (Fig. 3a–c). All mammalian cells have the capacity to synthesize cholesterol de novo from acetate in the endoplasmic reticulum, where HMGCR serves as the rate limiting enzyme. When sterols accumulate in the endoplasmic reticulum membrane, INSIG1 ubiquitinates HMGCR and facilitates its proteasomal degradation. Although the fold-change in expression of HMGCR and INSIG1 mRNA was less than 2, we were interested in determining whether the observed pattern was idiosyncratic to PPC1 cells or if it was observed in PGCC generated from other cancer cells. To address this question, we generated MEL624 melanoma PGCC and assessed changes in HMGCR and INSIG1 after LCL521 treatment. As in PPC1 prostate cancer cells, we detected a highly significant increase in INSIG1 mRNA and a smaller, but also significant increase in HMGCR following ASAH1 inhibition in PGCC (Fig. 3d).

### Cholesterol metabolism differs between parental cancer cells and PGCC

**Figure 4:**
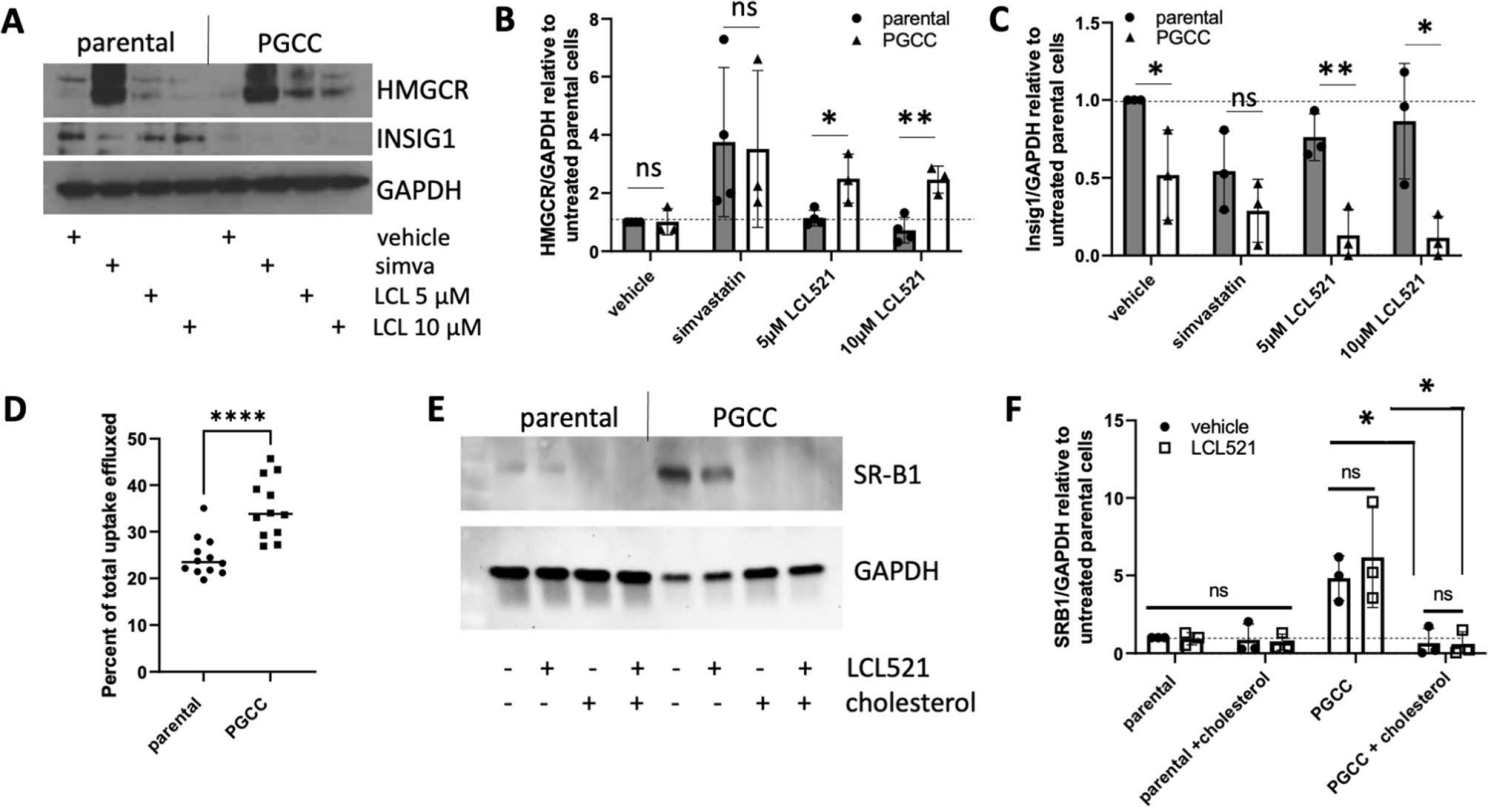
Cholesterol metabolism and trafficking in PGCC is upregulated. (**A**) Western blot analysis of HMCGR and INSIG1 after two days of treatment with 5 or 10 µM LCL521. Simvastatin (1 µM) was included as a positive control. Expression levels relative to GAPDH were calculated for both genes and normalized to untreated parental cells. Comparisons between parental and PGCC are shown for HMGCR (**B**) and INSIG1 (**C**), summarizing 3 or 4 independent experiments. (**D**) Quantification of cholesterol efflux, represented as percent released of total incorporated. (**E**) Western blot analysis of SR-B1, with 3 independent experiments quantified in (**F**). *p < 0.05; **p < 0.01, **** p < 0.0001.

Next, we investigated how inhibition of ASAH1 affects HMGCR and INSIG1 protein expression in parental cells and PGCC. Cholesterol synthesis is tightly regulated by negative feedback mechanism in order to conserve cellular energy and prevent toxicity. When levels of cholesterol are sufficient, INSIG1 protein promotes the degradation of HMGCR through activation of the sterol regulatory element-binding protein (SREBP). However, upon depletion of cholesterol, INSIG1 protein is rapidly degraded resulting in the release and nuclear translocation of SREBP, where it induces INSIG1 mRNA transcription^14^. Thus, mRNA and protein levels of INSIG1 are inversely correlated. To mimic cholesterol starvation, we treated cells with the HMCGR blocker simvastatin. As shown in Fig. 4a, HMGCR is barely detectable in parental cells or PGCC at baseline (lanes 1 and 5) but increases significantly with simvastatin treatment (lanes 2 and 6), indicating that cholesterol sensing is functional in both populations. When ASAH1 is inhibited by LCL521, HMGCR protein increases significantly in PGCC (lanes 7 and 8 pooled and compared to lane 5, p < 0.05) but not parental cells (lanes 3 and 4 pooled and compared to lane 1, p = 0.76). INSIG1 expression was not affected by ASAH1 inhibition in parental cells (p = 0.27) but was decreased in PGCC (p < 0.01). Quantification of gene expression is shown graphically in Fig. 4b,c to demonstrate changes in PGCC compared to relatively stable expression patterns of parental cells. Taken together, these results suggest that changes in HMGCR and INSIG1 mRNA in PGCC upon ASAH1 inhibition reflect that these cells sense a decrease in cholesterol levels.

Maintenance of cholesterol homeostasis is complex. In addition to de novo synthesis in the endoplasmic reticulum, cholesterol can be taken up from the extracellular environment, transported within cells via the endo-lysosomal network and, since most cells are not capable of efficiently catabolizing cholesterol, effluxed from the cell. When loaded with exogenous cholesterol and cultured in serum to mimic physiological conditions, PGCC efflux a higher proportion of cholesterol than parental cells (Fig. 4d). We also evaluated the expression of scavenger receptor, class B type 1 (SR-B1), a protein known to participate in both HDL-mediated cellular cholesterol uptake and efflux and found that compared to parental cells, PGCC express high levels of SR-B1and that treatment with exogenous cholesterol decreased SR-B1 expression in PGCC to levels detected in parental cells (Fig. 4e,f)^15^. LCL521 did not have a significant effect on the expression on SR-B1 (Fig. 4f). Overall, these results highlight differences in cholesterol metabolism between parental cancer cells and radiation-induced PGCC.

### Ceramide does not colocalize with cholesterol and accumulates in PGCC upon ASAH1 inhibition

**Figure 5:**
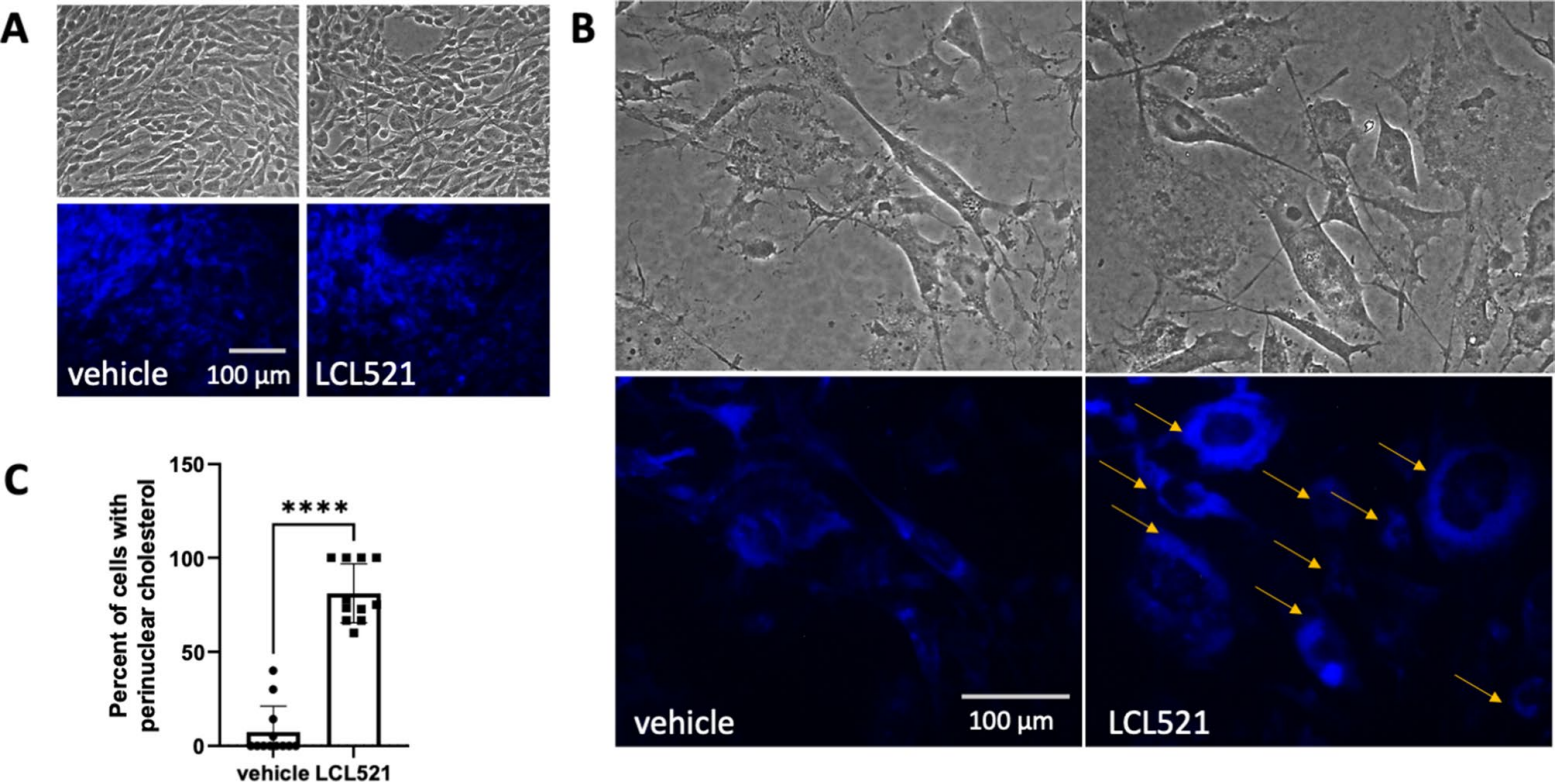
Cholesterol staining changes in PGCC upon LCL521 treatment. (**A**) Uncropped 20X images of parental cells stained with filipin III to visualize cholesterol after 48 h of indicated treatment. (**B**) 20X images of PGCC after the same protocol as (**A**). Yellow arrows indicate the perinuclear accumulation of cholesterol. (**C**) Quantification of at least three images per experiment over three experiments; vehicle treated PGCC n = 86 and LCL521 treated PGCC n = 73. **** p < 0.0001.

The endo-lysosomal network is important for distribution of cholesterol to other cellular organelles. Cholesterol is taken up as a complex with LDL and its receptor and is dissociated from the LDLR upon acidification in the endosomal lumen. The LDL-cholesterol is then transported to the late endosomes where hydrolysis by acid lipases releases free cholesterol, mediated by Niemann-Pick proteins. Defects in this process result in a lysosomal storage disorder that was recently shown to involve a cholesterol-dependent inverse relationship with ASAH1^16^. We therefore investigated how ASAH1 inhibition affects the intracellular distribution of cholesterol upon LCL521 treatment by staining cultures with filipin for visual evaluation^17^. Our results show that filipin staining is similar between untreated and LCL521 treated parental cells (Fig. 5a). In contrast, treatment of PGCC with LCL521 resulted in increased cholesterol staining in the perinuclear region (Fig. 5b,c), suggestive of disruption of cholesterol homeostasis. These results offer a potential explanation for the selective upregulation of HMGCR and INSIG1 mRNA in PGCC following ASAH1 inhibition.

**Figure 6:**
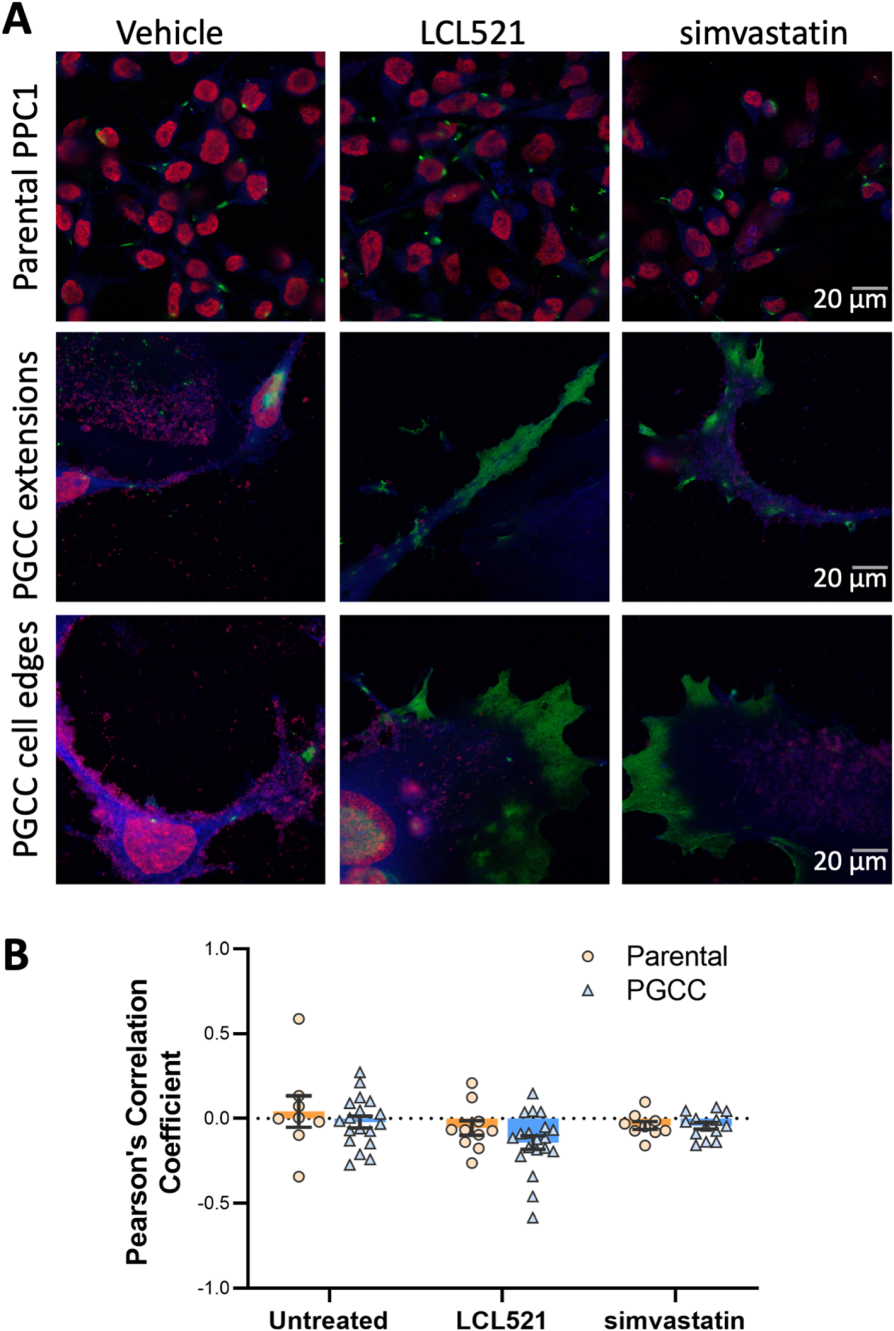
Ceramide and cholesterol segregate, with ceramide appearing at periphery of PGCC treated with LCL521 or simvastatin. (**A**) Uncropped 63X images were obtained of parental cells or PGCC treated with 5 µM LCL521 or 1 µM simvastatin for 48 h. Green indicates ceramide staining, which appears at cellular termini in treated PGCC. Blue indicates filipin staining of cholesterol and red indicates SYTOX deep red staining of nucleic acids. (**B**) Quantification of ceramide/cholesterol signal colocalization across all samples and conditions, showing a durable lack of colocalization for the lipids.

Inhibition of ASAH1 interferes with the hydrolysis of ceramide to sphingosine in the lysosome, leading to accumulation of ceramide. We previously demonstrated that inhibition of ASAH1 in PGCC either by LCL521 or off-target effect of tamoxifen results in a particular morphology that features long, thin extrusions that we termed “barren branches”^18^. As shown in Fig. 1, LCL521 treatment resulted in the accumulation of ceramide at the PGCC cell surface and in “barren branches” (Fig. S7). Since cholesterol is important for membrane fluidity and biophysical studies have shown that ceramide displaces cholesterol 1:1 in artificial membranes, we wanted to visualize the subcellular locations of ceramide and cholesterol in the absence or presence of LCL521 in the same cell^19^. Simvastatin was included as a control for cholesterol depletion. Treatment with LCL521 or simvastatin did not significantly impact parental cells (Fig. 6a upper row) but increased ceramide staining in PGCC within the cell extensions and cellular edges (Fig. 6a middle and lower rows). The average Pearson’s correlation for the untreated controls was − 0.055 ± 0.160. A one-sample t-test revealed no difference compared with 0 (t(df) = X, p = x), suggesting that these two lipids do not colocalize in the same membrane domain. Colocalization analysis of ceramide and cholesterol signals following treatment with LCL521 or simvastatin showed that the overall Pearson’s coefficient for the images remained stable across all treatment conditions with no significant differences detected with 2-way ANOVA, indicating the ceramide and cholesterol consistently exclude each other to the same degree, regardless of lipid levels (Fig. 6b). Ceramide staining intensity per cell increased significantly with LCL521 treatment (p < 0.001) but not with simvastatin (p = 0.12).

### Simvastatin treatment is sufficient to inhibit PGCC cell division

**Figure 7:**
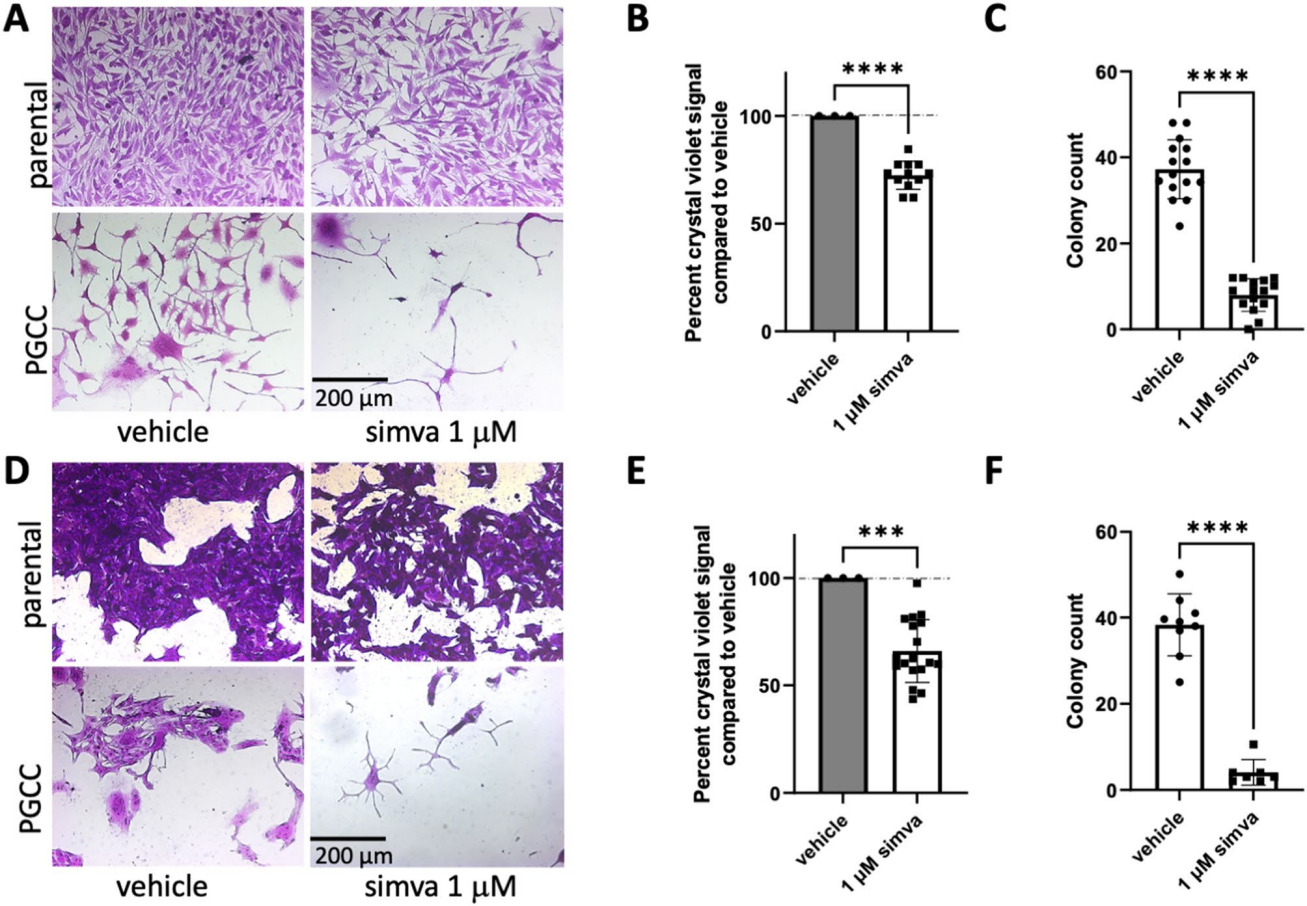
Simvastatin alone inhibits PGCC division. (**A**) 10X images of parental PPC1 cells or PGCC treated with DMSO or simvastatin. Daughter cells are shown mid-extrusion from DMSO treated PGCC while simvastatin treated PGCC lack progeny. (**B**) Growth inhibition in parental PPC1 exposed to simvastatin is quantified by the crystal violet viability assay, with DMSO treatment set at 100% viable. (**C**) Colony counts of filtered PGCC are quantified, with at least three independent experiments represented. (**D**) 10X images of parental MEL624 cells or their PGCC treated with DMSO or simvastatin. Statin-treated parental cells show slight growth inhibition as quantified in (**E**). Statin-treated PGCC show a loss of colony formation as quantified in (**F**), representing at least three independent experiments. ***p < 0.001, ****p < 0.0001.

Our results showed that ASAH1 inhibition leads to accumulation of ceramide at the cell surface and that ceramide and cholesterol do not colocalize. We therefore hypothesized that, since cholesterol is important for membrane fluidity, loss of cholesterol could affect membranes such that PGCC are unable to extrude progeny. To test whether lowering cholesterol has the same effect as inhibition of ASAH1 on PGCC progeny formation, we treated parental cells and PGCC with simvastatin and analyzed the effect on proliferation and colony formation, respectively. In parental cells, simvastatin exerted some growth inhibition likely because rapidly dividing cells require cholesterol to manufacture membranes for daughter cells (Fig. 7a,b). In PGCC simvastatin treatment resulted in “barren branching” as previously observed in response to ASAH1 inhibition (Fig. 7a, Fig. S7)^18^. Furthermore, simvastatin almost completely ablated the ability of PGCC to reproduce by primitive means as quantified through colony formation (Fig. 7c). Similar results were obtained in MEL624 melanoma cells, confirming that the effect of simvastatin on PGCC progeny and colony formation was not limited to PPC1 prostate cancer cells (Fig. 7d–f). These results indicate that inhibition of ASAH1 or interfering with cholesterol synthesis similarly inhibit the ability of PGCC to generate progeny.

## Discussion

It is clear from morphological observations that PGCC have undergone profound changes. Similar to our results shown in Fig. 2 other groups reported that PGCC have significantly altered gene expression^20,21^. Ribosomal genes and genes involved in DNA synthesis or cell cycle regulation were particularly impacted (Fig. 2). Downregulation of ribosomal genes and a concomitant increase in DNA synthesis genes may reflect that PGCC redirect efforts to DNA synthesis rather than protein synthesis. The increase in DNA synthesis genes in our study (suppl. Figure 3) contrasts with results in murine hepatocellular carcinoma cells (HEPA 1–6), which reported an overall downregulation in these genes^20^. This discrepancy could be related to timing, since we collected PGCC three days after exposure to irradiation, whereas the HEPA 1–6 derived population was analyzed after 14 days of continuous drug exposure. It is possible that a widespread upregulation of DNA synthesis genes, particularly in genes associated with the DNA synthesis-related portions of the cell cycle, reflect an initial period of DNA synthesis followed by a tapering with the reduced expression of DNA synthesis genes after 14 days of drug exposure. The Liu laboratory, which analyzed transcriptomes of untreated ovarian cancer cells, PGCC harvested after a single day of drug exposure and 3 days of recovery, and cells released from these PGCC, demonstrated sweeping differences between the populations along the experimental timeline^21^. Overall, the process of PGCC formation maybe more specific, delimited, and controlled than we currently understand. Thus, further temporal analysis is needed to gain a better understanding of the dynamics of DNA synthesis gene expression in PGCC that develop in response to stress exposure.

While the differences between the parental and PGCC populations deserve further analysis, we were primarily interested in understanding why LCL521, pro-drug that inhibits ASAH1 and minimally affects mitotically proliferating parental cells, nearly completely inhibits progeny formation in PGCC^13^. Consistent with our observation that LCL521 had no significant effects on the transcriptome of parental cells, this drug has no effect on primary tumor growth when used as a single agent in vivo^22^. In contrast, LCL521 is highly effective in preventing radiation therapy failure, which is why we were specifically interested in its effect on PGCC, which are induced by radiation stress^13,22^. RNA-seq analysis confirmed that even a treatment of only 5 h selectively resulted in significant increases of 4 transcripts in PGCC, including RRAGC, which assists in targeting MTOR to the lysosome^23^, and STX3, which facilitates secretory autophagy^24^. The exact roles of these genes in PGCC biology remain to be investigated but they could be affected downstream of ASAH1 inhibition by LCL521. In this study, we focused on HMGCR and INSIG1, since both genes are involved in the synthesis of cholesterol, which like sphingolipids, is found in cellular membranes, where it influences membrane fluidity and cell signaling.

Compared to parental cancer cells, PGCC-independent of ASAH1 inhibition- had lower expression of INSIG1 protein, more efficiently effluxed cholesterol, and expressed high levels of SR-B1 (Fig. 4). INSIG1 ubiquitinates HMGCR and facilitates its proteasomal degradation, thereby decreasing cholesterol synthesis. Reduced expression of INSIG1 in PGCC relative to parental cancer cells may reflect their higher need for cholesterol. PGCC can express stemness markers, demonstrate plasticity, are highly dedifferentiated, and have therefore been described as somatic equivalents of pre-implantation embryos^7^. Human blastomere (pre-implantation) and blastocyst (post-implantation) transcriptomes indicate that the former have highly enriched gene expression in cholesterol biosynthesis^25^. Thus, a decrease in INSIG1 protein reflects the altered cholesterol in PGCC compared to parental cancer cells, which represents another parallel between PGCC and the pre-implantation embryos.

SR-B1, which is increased in PGCC, is a multi-functional protein able to participate in both cholesterol efflux and uptake^26^. Aside from SR-B1 mediating the transfer of cholesterol between high density lipoproteins and normal cells, it also facilitates the uptake of cholesterol by malignant cells and can be considered an enabling factor for self-sufficiency in cancer^27,28^. In prostate cancer, SR-B1 is upregulated in both primary disease as well as in metastatic lesions of castrate resistant disease^29^. SR-B1 also participates in HDL-mediated cholesterol efflux, thus increased serum-induced cholesterol efflux observed in PGCC could be due to higher SR-B1 expression, as serum contains abundant amounts of HDL particles^26^. It should be noted that while SR-B1 protein was shown to be downregulated in cholesterol-loaded PGCC for immunoblotting, the cholesterol used for these experiments (MβCD:Chol) was different than the radiolabeled cholesterol used for the efflux experiments and this may have resulted in decreasing SR-B1 protein in MβCD:Chol-loaded PGCC due to the oversaturation of cellular cholesterol causing these cells to hinder cholesterol uptake through SR-B1 downregulation^30^. Moreover, it is possible that serum exposure to PGCC loaded with radiolabeled cholesterol may have upregulated SR-B1 protein in these cells, resulting in increased cholesterol efflux, or these cells may have increased serum-induced cholesterol efflux through other pathways^15^. However, as markers of PGCC have not been defined, increased SR-B1 could represent an additional candidate that in combination with other proteins could establish a panel of proteins suitable to identify PGCC in patient samples.

We observed that prolonged ASAH1 inhibition resulted in accumulation of cholesterol in the perinuclear region (Fig. 5), which differed from the cell surface accumulation of ceramide (Fig. 1). Hypothesizing that the accumulation could represent functional sequestration, we analyzed how parental cells and PGCC responded to cholesterol depletion using simvastatin and compared the response to ASAH1 inhibition. We found that inhibition of ASAH1 significantly increased HMGCR protein expression, which reflects a response of cells that sense low levels of cholesterol (Fig. 4). Reducing cholesterol has numerous potential effects in cancer cells, including diminished proliferation and impaired signaling via G protein-coupled receptors, MAPK, and Akt^31^. However, since cholesterol staining did not indicate that PGCC have low levels of this lipid upon ASAH1 inhibition, our results suggest that disruption of cholesterol trafficking may occur when ASAH1 function is inhibited. Thus, inhibition of ASAH1 in the lysosome appears to cause wide-ranging effects on lipid function in other compartments. Visualization of ceramide and cholesterol confirmed that the lipids do not colocalize and that ceramide levels were high at the edges of cells and in cytoplasmic extensions (Fig. 6). The mechanism by which ceramide accumulates at the cell surface following ASAH1 inhibition by LCL521 remains unclear and requires further investigation. One possibility is that ceramide increases as a result of sphingomyelinase activity. Radiation can directly stimulate neutral sphingomyelinase to generate ceramide in membranes and recently, neutral sphingomyelinase 2 (nSMase2, SMPD3) was found responsible for the generation of plasma membrane ceramide upon chemotherapy treatment^32,33^. Based on RNAseq expression data, PPC1 cells have low levels of SMPD3 but expression does increase significantly in PGCC (p = 0.00023). The expression of other neutral sphingomyelinases (SMPD2, SMPD4) is more robust but is either unchanged or decreased in PGCC. However, there is no plausible explanation on how ASAH1 inhibition would affect neutral sphingomyelinase activity at the plasma membrane. The expression of acid sphingomyelinase (SMPD1), which generates ceramide at the outer plasma membrane in response to various stresses, including radiation, is also unchanged between PPC1 cells and PGCC. However, like ASAH1, acid sphingomyelinase resides within lysosomes and it has recently been shown that radiation promotes the fusion of lysosomes with the plasma membrane followed by release of lysosomal content to the cell surface^34^. Thus, it is plausible that the visualized increase in surface ceramide in LCL521 treated PGCC is contained within lysosomes that are being translocated to and/or fusing with the plasma membrane. Since simvastatin did not significantly increase total ceramide levels in the cell, our data suggest that the localized increase in ceramide upon drug treatment of PGCC may occur as a consequence of altered cholesterol metabolism. Our results cannot distinguish whether cholesterol failed to be transported to the plasma membrane or if ceramide displaced the lipid. Displacement of cholesterol by ceramide has been documented and appears to depend on the relative ratios of the molecules^35–37^. Cholesterol can cause dynamic changes in membrane curvature and plays important roles in exocytosis^38^. Similarities between mechanisms underlying exocytosis and budding of PGCC progeny remain to be defined but the “barren branching” morphology and our previous analysis by time-lapse microscopy are supportive of the hypothesis that membranes of PGCC treated with LCL521 are stiffer than those of untreated PGCC. Since cholesterol depletion resulted in similar morphology as ASAH1 inhibition in PGCC, we hypothesized that treatment with simvastatin should impair PGCC progeny formation. Our data supported this hypothesis, but further biophysical measurements would be required to directly demonstrate membrane stiffness.

The novel observation that simvastatin inhibits PGCC progeny formation is interesting because numerous studies have demonstrated benefits of lipophilic statins in cancer patients^39,40^. Unlike hydrophilic statins that act primarily in the liver, lipophilic statins are disseminated throughout the body, which may explain their benefits in cancer patients. Hypotheses behind the benefits of statins in cancer include downregulation of EMT markers, a decline in MYC expression, and preferential action against “stem-like” subpopulations^41–44^. Simvastatin, the most lipophilic statin, has been associated with a decrease in cancer recurrence in multiple clinical studies^45–47^. A meta-analysis of 30 studies investigating statin use in prostate cancer patients revealed that statin use lowered recurrence rates and increased survival specifically in patients who received radiation therapy as it did not reduce the incidence of prostate cancer development or affect recurrence rates or survival in patients who underwent surgical removal of the prostate^48^. This radiation therapy specific effect is interesting in the context of PGCC, since this population is promoted by stress and our current results show that progeny generation is inhibited by simvastatin. Our results support the possibility that benefits of simvastatin in radiation-treated prostate cancer patients or LCL521 in a preclinical model of prostate cancer during radiation therapy inhibited PGCC progeny formation and contributed to improved outcomes^22,48^. Our data suggest that dependence of PGCC on cholesterol is a vulnerability that is highly actionable and could be quickly leveraged in the clinic by placing patients on simvastatin during radiation or chemotherapy.

## Materials and methods

### Cell lines, generation of PGCC, and reagents

PPC1 prostate cancer and MEL624 melanoma cells were kind gifts from Dr. Dean Tang (Roswell Park Comprehensive Cancer Center, Buffalo, NY) and Dr. Michael Nishimura (Loyola University, Chicago, IL), respectively. All cells were cultured in RPMI-1640 medium (Cellgro, Manassas, VA), Antibiotic–Antimycotic (1%; Gibco), and FBS (10%; Hyclone). Cells were maintained at 37 °C with 5% CO_2_. PGCC were generated by exposing parental cells, plated 8 × 10^5^/100 mm dish overnight, to a single 8 Gy dose of gamma irradiation (^137^Cs γ‐irradiator, J.L Sheperd & Associates) or 5 nM docetaxel (Zydus Hospira Oncology). On day 3, PGCC were captured through size exclusion filtration using a 20 micron cell strainer (PluriSelect), which resulted in a highly enriched PGCC working populations (Fig. S8). LCL521 was synthesized at the MUSC Lipidomics Core and reconstituted in 100% ethanol to 50 mM^49,50^. Simvastatin was obtained from Cayman Chemicals (#10010344).

### RNA-sequencing

PPC1 cells were allowed to attach overnight. The next day, half of the plates were irradiated (8 Gy) to generate PGCC. On day 3, cells were exposed to 5 μM LCL521 for 5 h and were then detached by trypsinization. Cells from irradiated plates were filtered to enrich for PGCC. RNA was isolated (Qiagen RNAeasy kit) and quantity and purity verified (Nanodrop from Thermo Fisher). A total of 6 RNA samples per condition were submitted to Novogene (Cambridge, UK). Prior to library construction, Novogene repeated verification RNA integrity (Agilent 2000). Workflows for generation of the library and subsequent bioinformatic analysis by Novogene are shown in Fig. S9. Downstream analysis was performed using a combination of programs including STAR, HTseq, Cufflink and our wrapped scripts. Alignments were parsed using Tophat program and differential expressions were determined through DESeq2. GO and KEGG enrichment were implemented by the ClusterProfiler^51–59^. For additional details please see supplementary methods.

### Real time-PCR

MEL624 cells were exposed to 8 Gy and treated with vehicle or LCL521 (5 µM) for 5 h on day 3. RNA was extracted from PGCC (RNAeasy kit, Qiagen) and reverse transcribed (Promega kit, #A5000), and qPCR was performed with iQSYBR Green master mix (BioRad, catalog #1708882) using a CFX Connect Real-time PCR Detection System (Bio-Rad, MUSC Proteogenomic facility). PCR primers were from realtimeprimers.com (Elkins Park, PA). The HMGCR primer set: Forward 5'- CTT GGT TTT TGG CTC TTT CA -3', Reverse 5'- GTC AAT TGC ACT GAT CAC CA -3'. The INSIG1 primer set: Forward 5'- TGG CCT ACT GTA CCC CTG TA -3', Primer 5'- GGA CAG CTG GAC ATT ATT GG -3'.

### Immunoblotting

For analysis of HMGCR and INSIG1, PPC1 cells were plated and PGCC induced by radiation stress. On day 3, media was refreshed, and cells treated with vehicle, 1 µM simvastatin, 5 µM LCL521, or 10 µM LCL521. Media and treatments were refreshed again at 24 h and samples collected at 48 h. For analysis of SR-B1, PPC1 cells were exposed to vehicle or 5 nM docetaxel to generate PGCC. After 72 h, cells were washed with PBS, and each set of cells were treated with one of the following conditions: (1) vehicle only in serum-free medium; (2) LCL521 (5 µM) in serum-free medium; (3) serum-free medium containing the water-soluble cholesterol, cholesterol–methyl-β-cyclodextrin (MβCD:Chol) (10 µg/mL; Sigma-Aldrich, St. Louis, MO); (4) LCL521 (5 μM) in serum-free medium containing MβCD:Chol (10 µg/mL). Twenty-four hours after treatments, cells were washed with PBS, and respective treatments re-initiated for another 24 h. Cells not exposed to docetaxel were washed with PBS, and then cells harvested using RIPA lysis buffer containing mammalian protease inhibitors (VWR Life Science). PGCC were captured by size exclusion filtration and then lysed in RIPA buffer containing mammalian protease inhibitors. Protein was quantified and equal amounts of protein were separated by SDS-PAGE, transferred to nitrocellulose or PVDF membranes, blocked, and incubated with the following primary antibodies: anti-HMGCR (1:1,000, sc-271595), anti-INSIG1 (1:1,000, sc-390504), or anti-GAPDH (1:1,000, sc-32233), all from (Santa Cruz Biotechnology) or SR-B1 (1:1,000, MABC730; EMD Millipore). Secondary antibodies were 1:50,000 goat anti-mouse IgG-HRP (#7076S, Cell Signaling), goat anti-mouse IgM-HRP or goat anti-mouse IgG-HRP (1:10,000,Sigma-Aldrich). ECL substrate (Immobilon ECL Ultra Western HRP Substrate; Millipore Sigma) was used for protein detection. Film or a ChemiDoc machine (Analytik Jena US) were used for visualization of signals. Band intensity was quantified using ImageJ (open source, NIH) and the results expressed as the ratio of protein-of-interest/GAPDH. Data were normalized to untreated parental cells. Full images of all blots are shown in Fig. S10 and 11.

### Cholesterol efflux assay

PPC1 cells were allowed to grow to 50–60% confluency and treated with vehicle or 5 nM docetaxel for 72 h. Cells were trypsinized and PGCC from docetaxel treated cultures were enriched by filtration before plating vehicle treated and PGCC evenly onto a 48-well tissue culture plate. After 24 h, cells were washed with PBS, and then cholesterol-loaded via using serum-free media containing [^3^H] cholesterol (1 μCi/mL; PerkinElmer). After 24 h, cells were washed and treated with vehicle only or 5 μM of LCL521 in serum-free medium for 4 h. After treatments, cells were washed with PBS, and then incubated with human serum (2.5%; Sigma-Aldrich) diluted in serum-free medium for 4 h. Medium and cells were then collected/harvested and [^3^H] were counted in medium/cells as described^60^. Percent serum-induced cholesterol efflux was calculated by dividing the amount of [^3^H] in the medium by the amount of [^3^H] in cells plus medium and multiplying by 100.

### Confocal microscopy

Cells were plated into one row each on an 8 well chamber slide (#177445, Nalge-Nunc, Lab-Tek) to a confluency of 30%. After treatment cells were washed, fixed with 4% paraformaldehyde for 5 min, permeabilized with 0.01% Triton-X for 5 min, incubated overnight with anti-ceramide primary antibody 1:50 (#ALX-804-196-T050, Enzo), washed and incubated with anti-mouse FITC-conjugated secondary at 1:100 (#sc-2010, Santa Cruz) for one hour. After washing, cells were cover-slipped using ProLong Diamond anti-fade with DAPI (#P36962, Invitrogen). Signals were imaged by a Zeiss LSM 880 NLO inverted laser scanning confocal microscope using a 63X 1.4 N.A. planapochromat oil immersion lens.

Alternatively, after removal of primary anti-ceramide antibody, cells were incubated with Filipin III (#70440, Cayman Chemicals), and anti-mouse FITC-conjugated secondary at 1:50 (#sc-2010, Santa Cruz). Slides were mounted using ProLong Diamond antifade with SYTOX (Invitrogen). For cholesterol localization, at least 9 images from three experiments were captured for each condition using a UV laser at 20X on a Zeiss Axiovert 200. PGCC with clear peri-nuclear staining are expressed as a percentage of all cells per field. For cholesterol and ceramide colocalization, images (n = 5–6/group) of parental cells and PGCC were acquired using a Carl Zeiss LSM 880 confocal microscope (pixel size: 132 × 132 nm; 2 frame average) using a 63 × oil immersion objective (Plan-Apochromat, Carl Zeiss, NA = 1.4, working distance = 190 µm) and 405, 488, and 561 nm laser lines. Raw images were deconvolved in AutoQuant X (version 2.2.2; Media Cybernetics, Rockville, MD, USA) using the automatic filter strength setting. The CoLoc module in Imaris XT (version 9.1; Bitplane; Zurich, Switzerland) was used to generate intensity-based colocalization analysis between ceramide and cholesterol expression following previous methods^61^. Thresholds were first set individually for each channel using the automated function within the CoLoc module, and the Pearson’s correlation coefficient was determined for the ceramide and cholesterol channels. Quantification of ceramide/PGCC was achieved by dividing the signal intensity value by the cell count to obtain an average signal/cell.

### Viability crystal violet assay and colony count assays

Assays were performed as previously described^13^. Results are represented as percent signal in simvastatin-treated samples relative to vehicle treated samples. Colonies formation from PGCC in the 6 well plates was assessed visually by counting at 5 × magnification.

### Statistical analysis

RNA-seq data was collected from 6 sets of RNA isolations and analysis was performed by Novogene. All other experiments were performed with 3 technical and at least 3 biological replicates with all individual data points represented in summary graphs. Data were analyzed using Graph Pad Prism 9, with p < 0.05 considered significant. Multi-sample sets were first evaluated with the appropriate ANOVA test; Student’s t-test was used to test specific comparison hypotheses.

## Supplementary Information

Supplementary Information.

## Data Availability

All data generated and analyzed during the current study are included in this published article (and Supplementary Information files). The datasets generated and analyzed during the current study are available in the Gene Expression Omnibus (GEO) database repository accession GSE195919. https://www.ncbi.nlm.nih.gov/geo/query/acc.cgi?acc=GSE195919.
