# Supplementary material for: Polyploid giant cancer cells are dependent on cholesterol for progeny formation through amitotic division
Source: Sci Rep. 2022 May 27;12:8971. doi: 10.1038/s41598-022-12705-4 (PMC9142539; doi:10.1038/s41598-022-12705-4)
Supplement: Supplementary file 1 — Supplementary Information. [file 41598_2022_12705_MOESM1_ESM.pdf]

Polyploid Giant Cancer Cells are dependent on cholesterol for progeny formation through amitotic division

Shai White-Gilbertson[1], Ping Lu[1], Ikechukwu Esobi[2], Jing Echesabal-Chen[2], Patrick J. Mulholland[3], Monika Gooz[4], Besim Ogretmen[5], Alexis Stamatikos[2], Christina Voelkel-Johnson[1,5]*

[1] Medical University of South Carolina, Department of Microbiology and Immunology
[2] Clemson University, Department of Food, Nutrition, and Packaging Sciences
[3] Medical University of South Carolina, Department of Neuroscience, Charleston Alcohol Research Center
[4] Medical University of South Carolina, Department of Drug Discovery & Biomedical Sciences
[5]Medical University of South Carolina, Department of Biochemistry and Molecular Biology
*Corresponding author: johnsocv@musc.edu, Medical University of South Carolina, Basic Science Building, MSC250504, 173 Ashley Ave., Charleston, SC, USA. Tel: +1843-792-3125; Fax +1843-792-2464

Supplementary Information

Supplementary Methods

Data Analysis. Downstream analysis was performed using a combination of programs including STAR, HTseq, Cufflink and our wrapped scripts. Alignments were parsed using Tophat program and differential expressions were determined through DESeq2. GO and KEGG enrichment were implemented by the ClusterProfiler (1-9).

Reads mapping to the reference genome. Reference genome and gene model annotation files were downloaded from genome website browser (NCBI/UCSC/Ensembl) directly. Indexes of the reference genome was built using STAR and paired-end clean reads were aligned to the reference genome using STAR (v2.5). STAR used the method of Maximal Mappable Prefix (MMP) which can generate a precise mapping result for junction reads.

Quantification of gene expression level. HTSeq (v0.6.1) was used to count the read numbers mapped of each gene. And then FPKM of each gene was calculated based on the length of the gene and reads count mapped to this gene. FPKM, Reads Per Kilobase of exon model per Million mapped reads, considers the effect of sequencing depth and gene length for the reads count at the same time, and is currently the most commonly used method for estimating gene expression levels (10).

Differential expression analysis. Differential expression analysis between two conditions/groups (two biological replicates per condition) was performed using the DESeq2 R package (2_1.6.3). DESeq2 provide statistical routines for determining differential expression in digital gene expression data using a model based on the negative binomial distribution. The resulting P-values were adjusted using the Benjamini and Hochberg's approach for controlling the False Discovery Rate (FDR). Genes with an adjusted P-value <0.05 found by DESeq2 were assigned as differentially expressed. The Venn diagrams were prepared using the function vennDiagram in R based on the gene list for different group.

Correlations. To allow for log adjustment, genes with 0 FPKM are assigned a value of 0.001. Correlation were determined using the cor.test function in R with options set alternative =”greater” and method= “Spearman”

Clustering. To identify the correlation between difference, we clustered different samples using expression level FPKM to see the correlation using hierarchical clustering distance method with the function of heatmap, SOM(Self-organization mapping) and kmeans using silhouette coefficient to adapt the optimal classification with default parameter in R.

GO and KEGG enrichment analysis of differentially expressed genes. Gene Ontology (GO) enrichment analysis of differentially expressed genes was implemented by the clusterProfiler R package, in which gene length bias was corrected. GO terms with corrected P-value less than 0.05 were considered significantly enriched by differential expressed genes. KEGG is a database resource for understanding high-level functions and utilities of the biological system, such as the cell, the organism and the ecosystem, from molecular level information, especially large-scale molecular datasets generated by genome sequencing and other high-through put experimental technologies (http://www.genome.jp/kegg/). We used cluster Profiler R package (v.2.4.3) to test the statistical enrichment of differential expression genes in KEGG pathways.

Differentially expressed gene annotation. TFCat and Cosmic database were used to annotate the differential expressed gene. TFCat is a curated catalog of mouse and human transcription factors (TF) based on a reliable core collection of annotations obtained by expert review of the scientific literature. COSMIC is a database designed to store and display somatic mutation information and related details which contains information relating to human cancers.

References

1. Cock PJ, Fields CJ, Goto N, Heuer ML, Rice PM. The Sanger FASTQ file format for sequences with quality scores, and the Solexa/Illumina FASTQ variants. Nucleic Acids Res. 2010;38(6):1767-71. Epub 2009/12/18. doi: 10.1093/nar/gkp1137. PubMed PMID: 20015970; PMCID: PMC2847217.
2. Dobin A, Davis CA, Schlesinger F, Drenkow J, Zaleski C, Jha S, Batut P, Chaisson M, Gingeras TR. STAR: ultrafast universal RNA-seq aligner. Bioinformatics. 2013;29(1):15-21. Epub 2012/10/30. doi: 10.1093/bioinformatics/bts635. PubMed PMID: 23104886; PMCID: PMC3530905.
3. Erlich Y, Mitra PP, delaBastide M, McCombie WR, Hannon GJ. Alta-Cyclic: a self-optimizing base caller for next-generation sequencing. Nat Methods. 2008;5(8):679-82. Epub 2008/07/08. doi: 10.1038/nmeth.1230. PubMed PMID: 18604217; PMCID: PMC2978646.
4. Finn RD, Tate J, Mistry J, Coggill PC, Sammut SJ, Hotz HR, Ceric G, Forslund K, Eddy SR, Sonnhammer EL, Bateman A. The Pfam protein families database. Nucleic Acids Res. 2008;36(Database issue):D281-8. Epub 2007/11/28. doi: 10.1093/nar/gkm960. PubMed PMID: 18039703; PMCID: PMC2238907.
5. Jiang L, Schlesinger F, Davis CA, Zhang Y, Li R, Salit M, Gingeras TR, Oliver B. Synthetic spike-in standards for RNA-seq experiments. Genome Res. 2011;21(9):1543-51. Epub 2011/08/06. doi: 10.1101/gr.121095.111. PubMed PMID: 21816910; PMCID: PMC3166838.
6. Anders S, Huber W. Differential expression analysis for sequence count data. Genome Biol. 2010;11(10):R106. Epub 2010/10/29. doi: 10.1186/gb-2010-11-10-r106. PubMed PMID: 20979621; PMCID: PMC3218662.
7. Yu G, Wang LG, Han Y, He QY. clusterProfiler: an R package for comparing biological themes among gene clusters. OMICS. 2012;16(5):284-7. Epub 2012/03/30. doi: 10.1089/omi.2011.0118. PubMed PMID: 22455463; PMCID: PMC3339379.
8. Kanehisa M, Goto S. KEGG: kyoto encyclopedia of genes and genomes. Nucleic Acids Res. 2000;28(1):27-30. Epub 1999/12/11. doi: 10.1093/nar/28.1.27. PubMed PMID: 10592173; PMCID: PMC102409.
9. Altschul SF, Madden TL, Schaffer AA, Zhang J, Zhang Z, Miller W, Lipman DJ. Gapped BLAST and PSI-BLAST: a new generation of protein database search programs. Nucleic Acids Res. 1997;25(17):3389-402. Epub 1997/09/01. doi: 10.1093/nar/25.17.3389. PubMed PMID: 9254694; PMCID: PMC146917.
10. Mortazavi A, Williams BA, McCue K, Schaeffer L, Wold B. Mapping and quantifying mammalian transcriptomes by RNA-Seq. Nat Methods. 2008;5(7):621-8. Epub 2008/06/03. doi: 10.1038/nmeth.1226. PubMed PMID: 18516045.

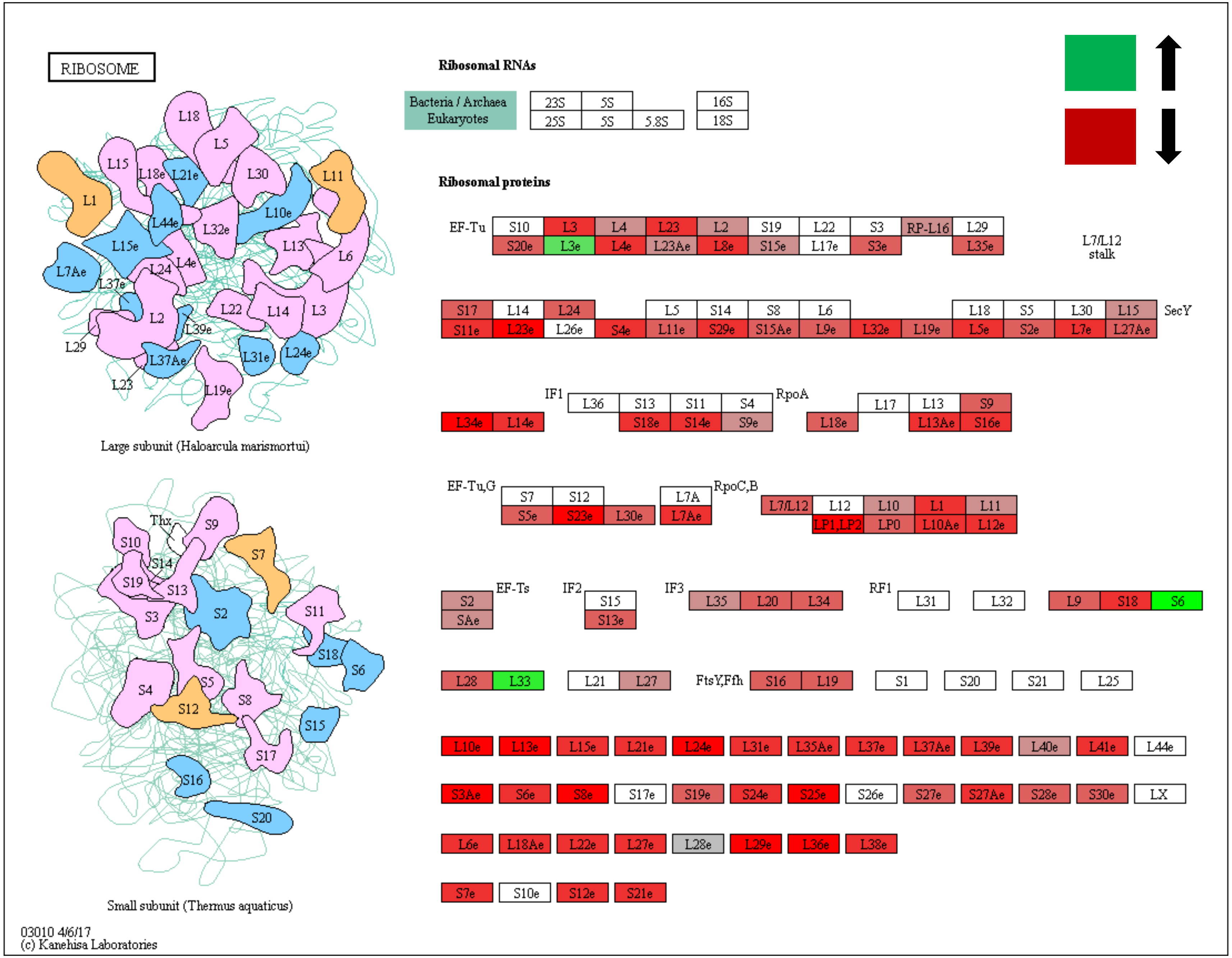

Figure S1. Ribosomal genes in PGCC relative to parental cells. This illustration was generated by Novogene using ClusterProfiler v2.4.3.

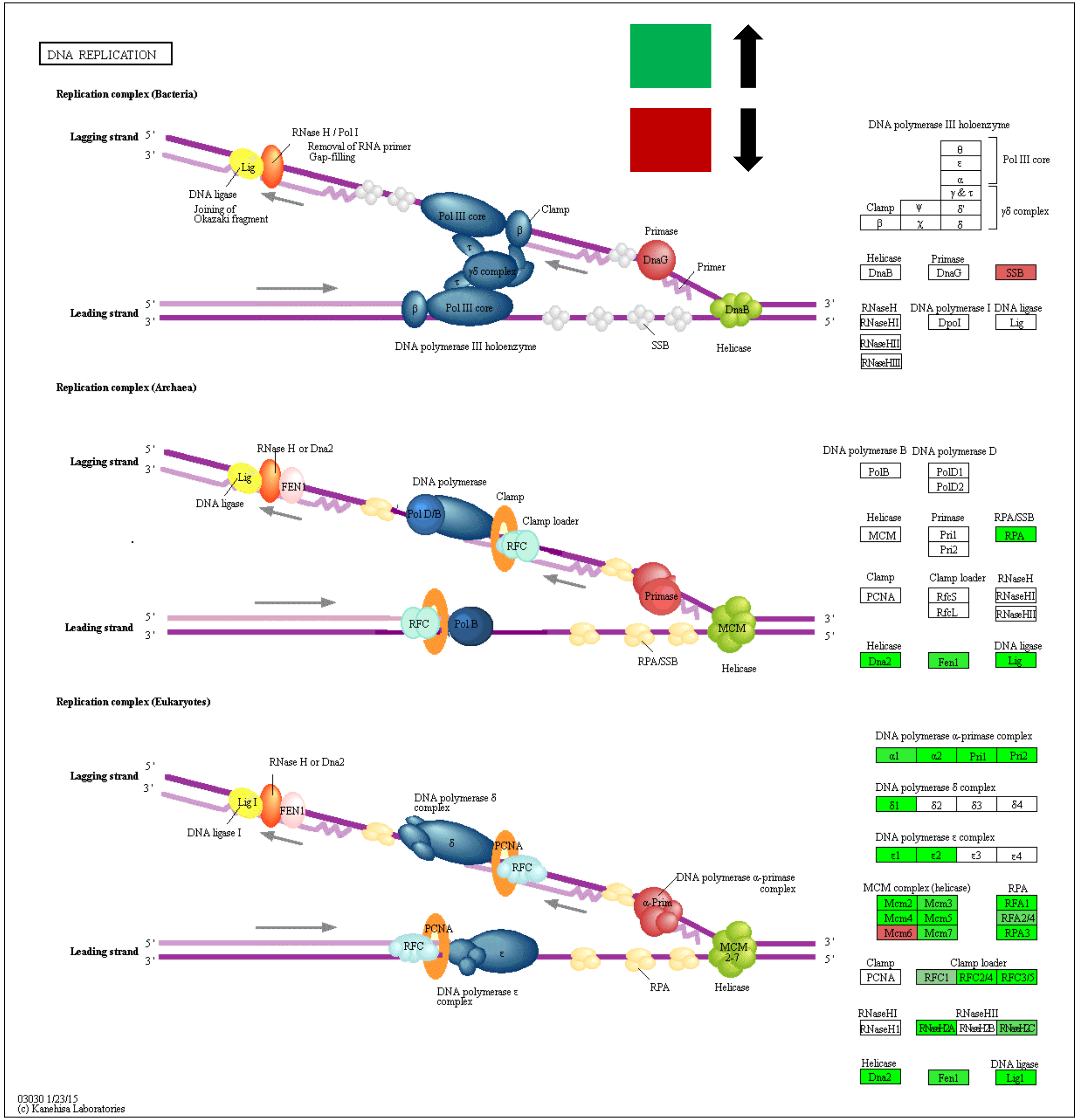

Figure S2. DNA replication genes in PGCC relative to parental cells. This illustration was generated by Novogene using ClusterProfiler v2.4.3.

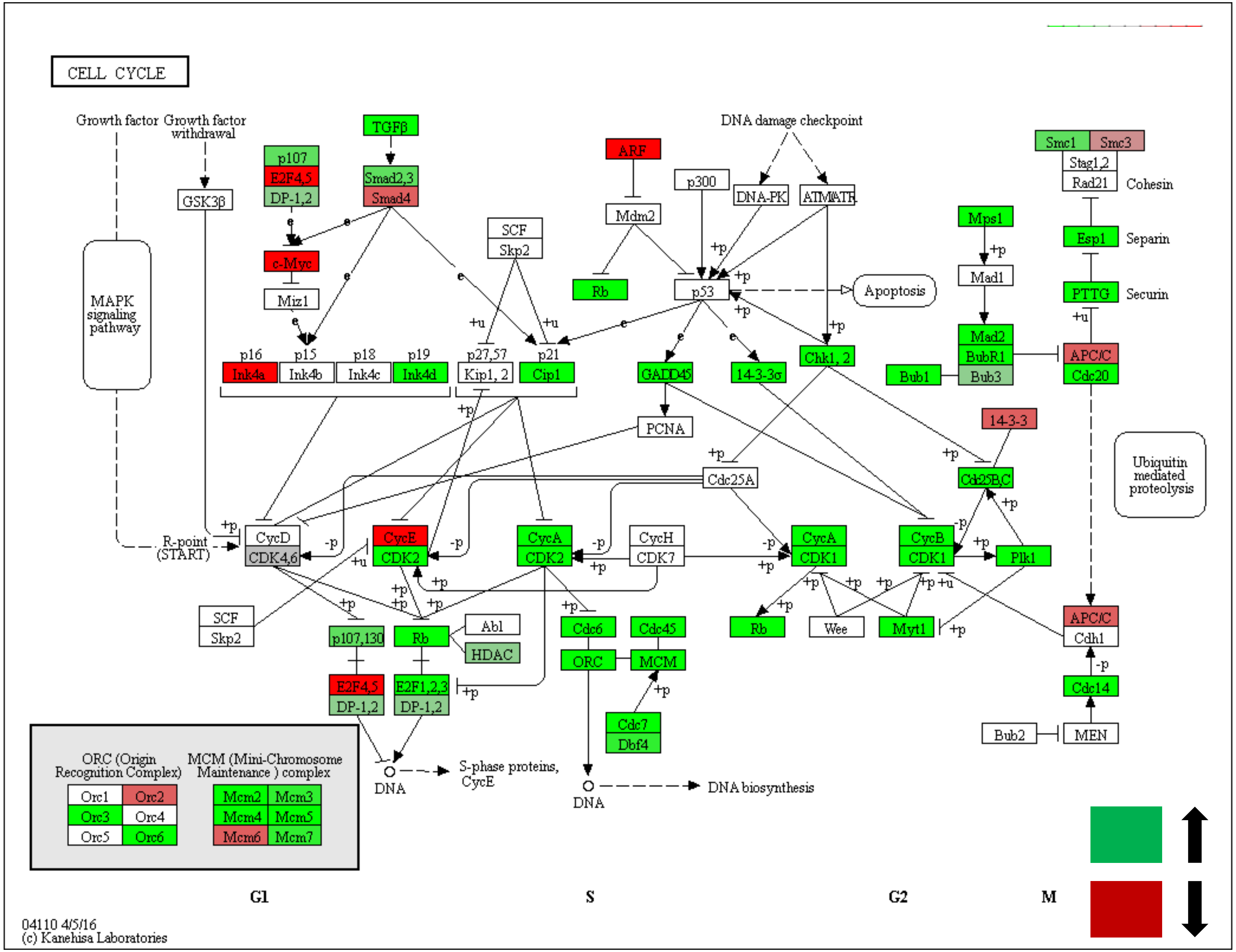

Figure S3. Cell cycle genes in PGCC relative to parental cells. This illustration was generated by Novogene using ClusterProfiler v2.4.3.

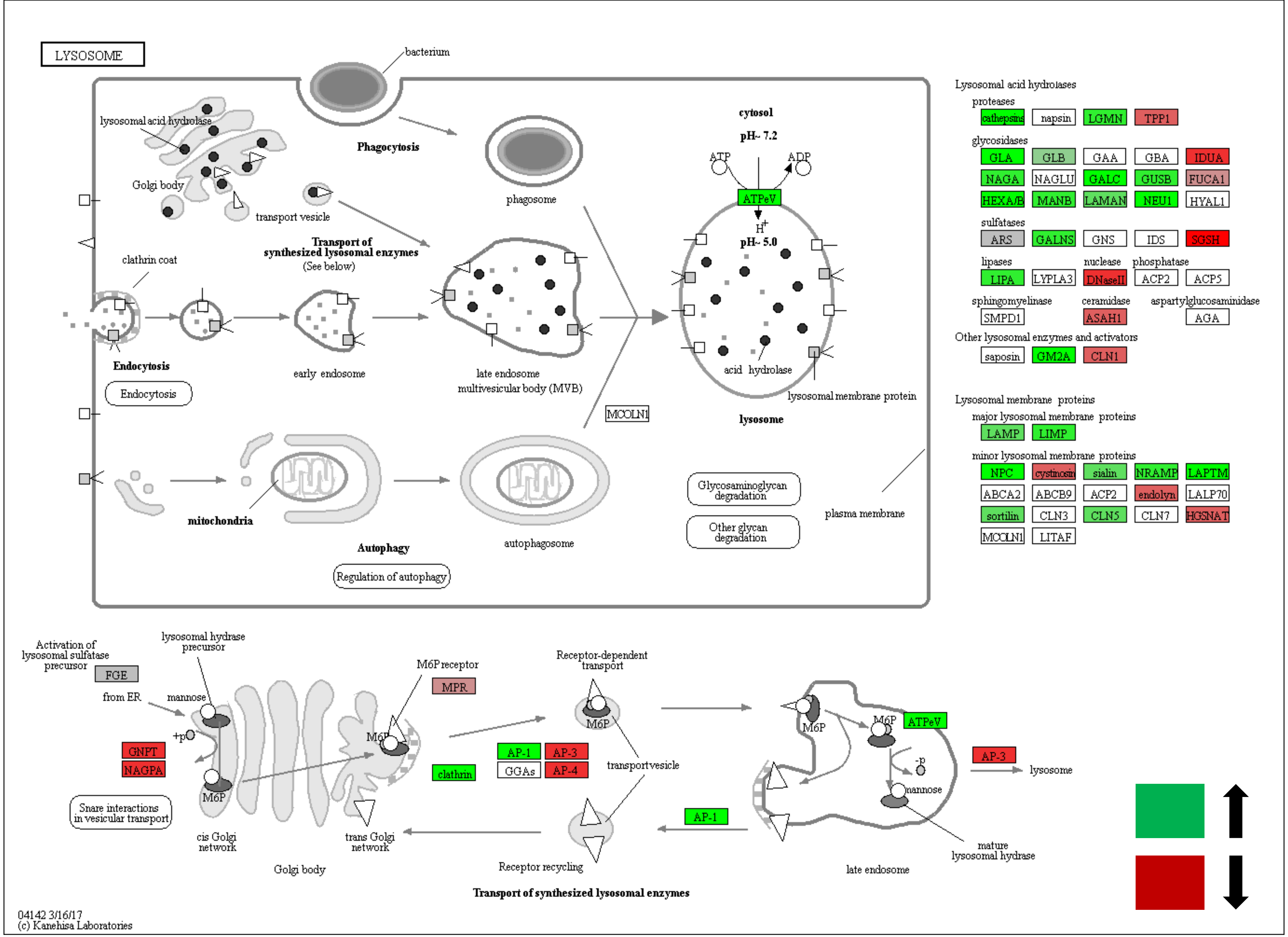

Figure S4. Lysosomal genes in PGCC relative to parental cells. This illustration was generated by Novogene using ClusterProfiler v2.4.3.

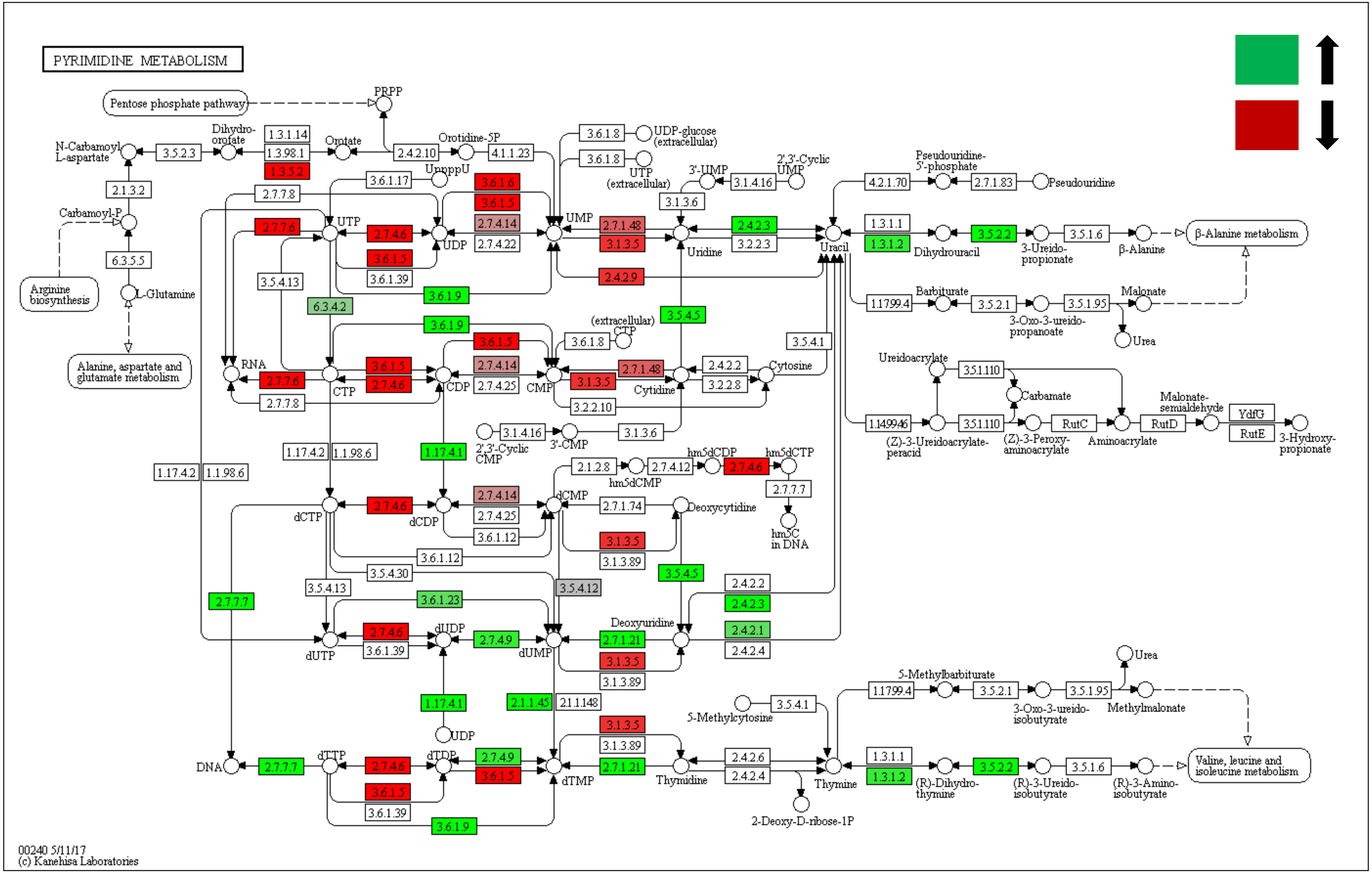

Figure S5. Pyrimidine metabolism genes in PGCC relative to parental cells. This illustration was generated by Novogene using ClusterProfiler v2.4.3.

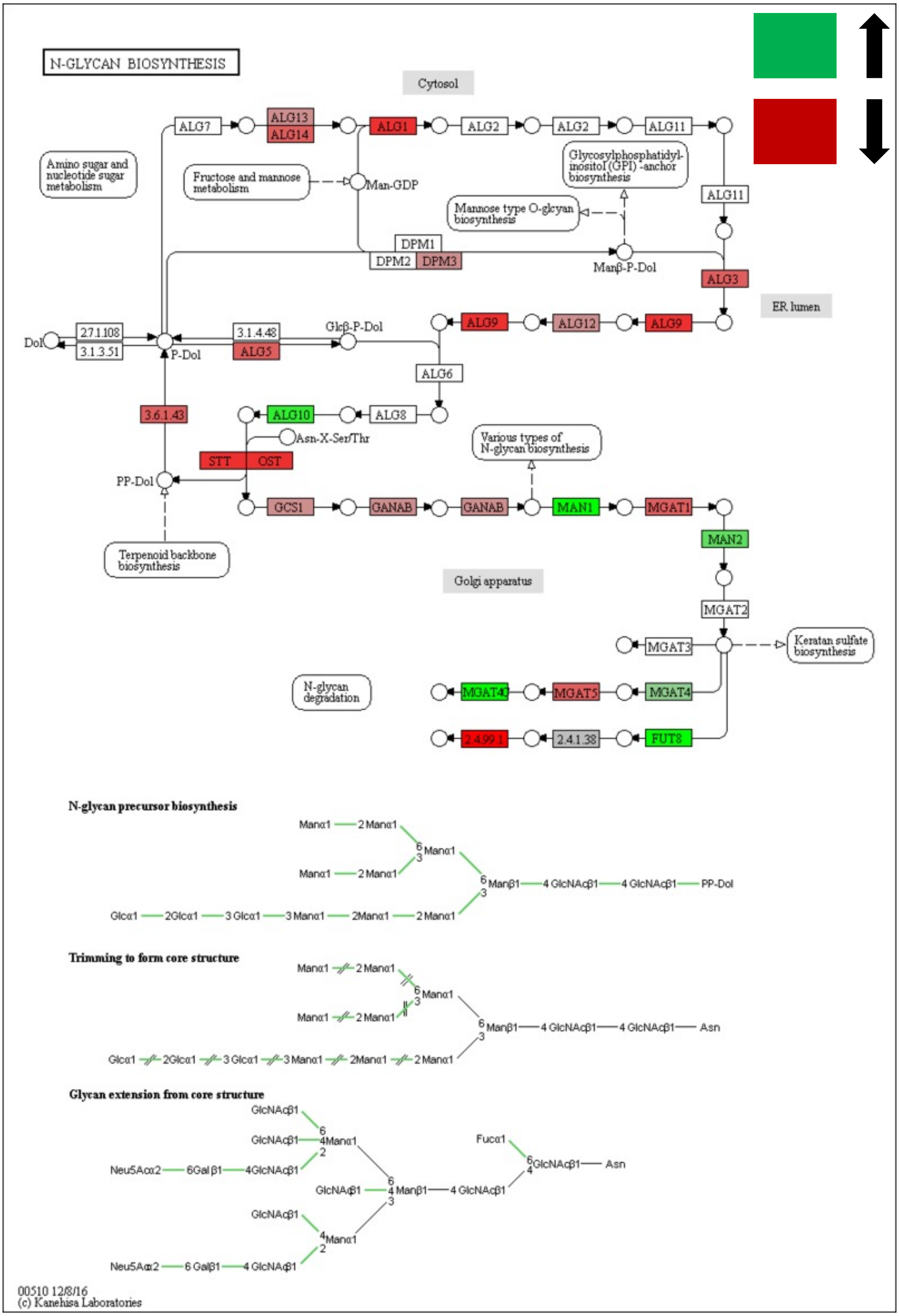

Figure S6. N-glycan synthesis in PGCC relative to parental cells. This illustration was generated by Novogene using ClusterProfiler v2.4.3.

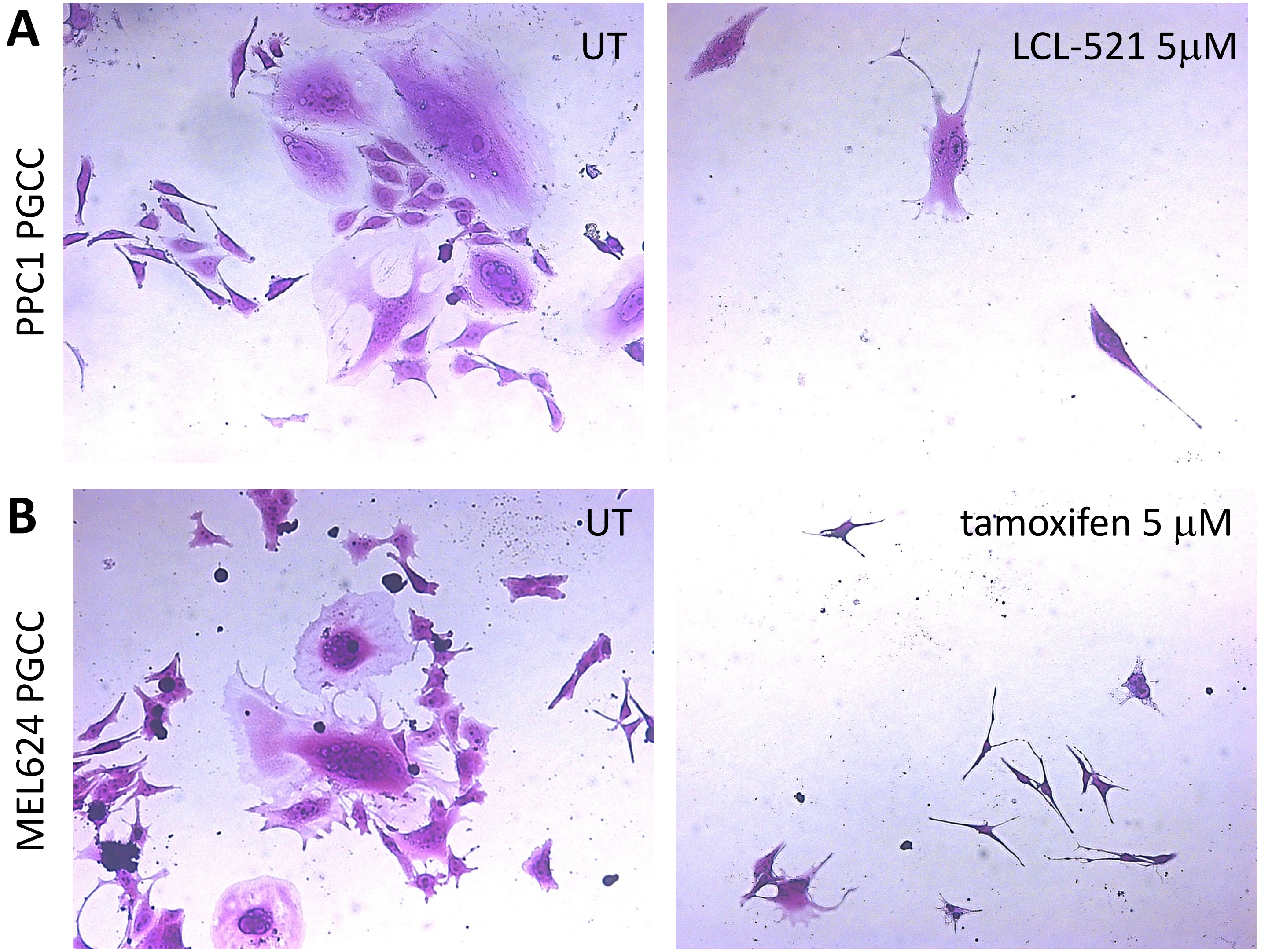

Figure S7. "Barren branching" morphology Uncropped 10X images of PGCC extruding progeny when left untreated or extending thin projections without daughter cells upon inhibition of ASAH1 with 5 μM of either LCL-521 (A) or 4-OH tamoxifen (Calbiochem/Sigma #579002) (B).

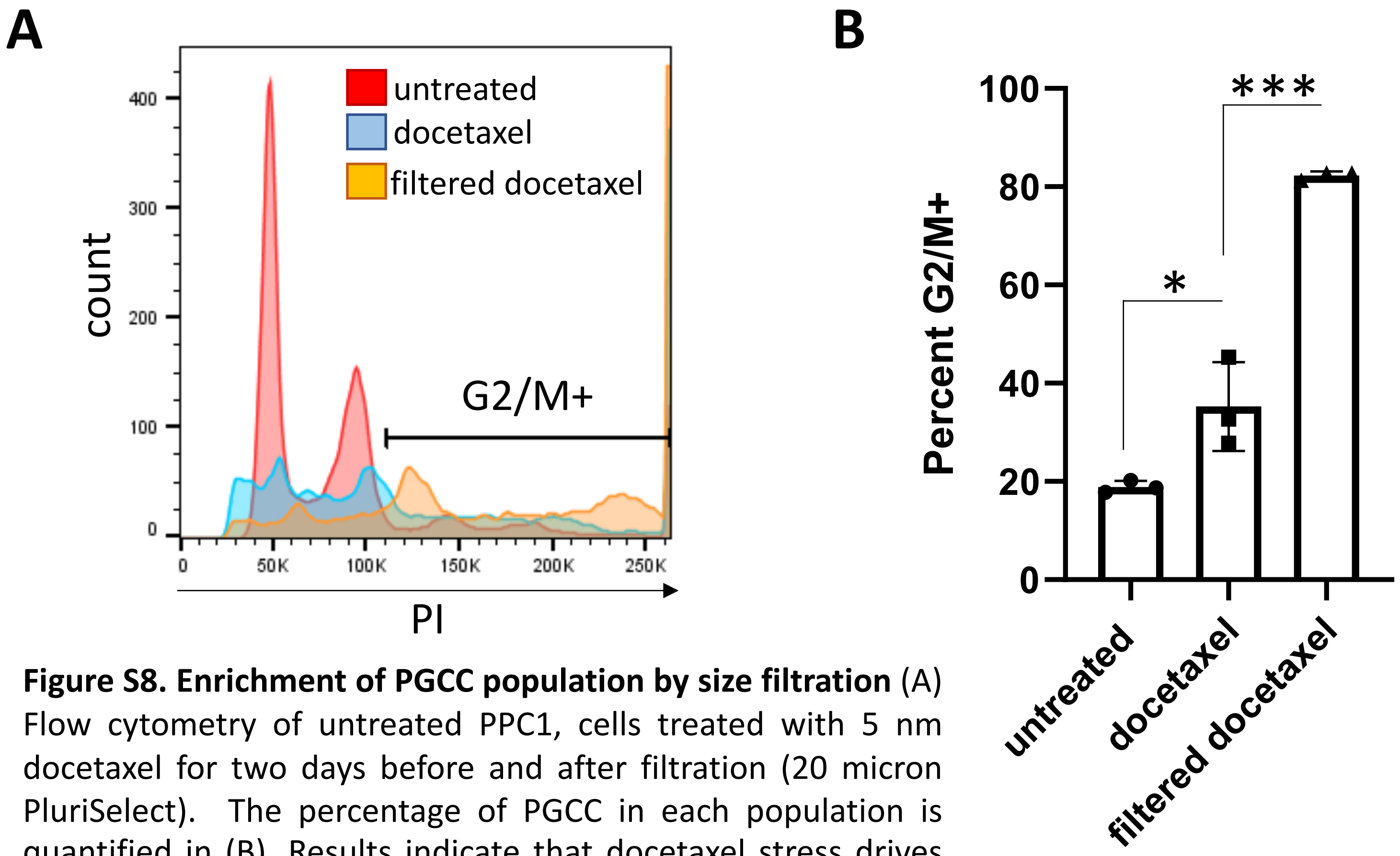

Figure S8. Enrichment of PGCC population by size filtration (A) Flow cytometry of untreated PPC1, cells treated with 5 nm docetaxel for two days before and after filtration (20 micron PluriSelect). The percentage of PGCC in each population is quantified in (B). Results indicate that docetaxel stress drives PGCC formation, * $p<0.05$ and that filtration results in highly enriched PGCC, *** $p<0.00001$.

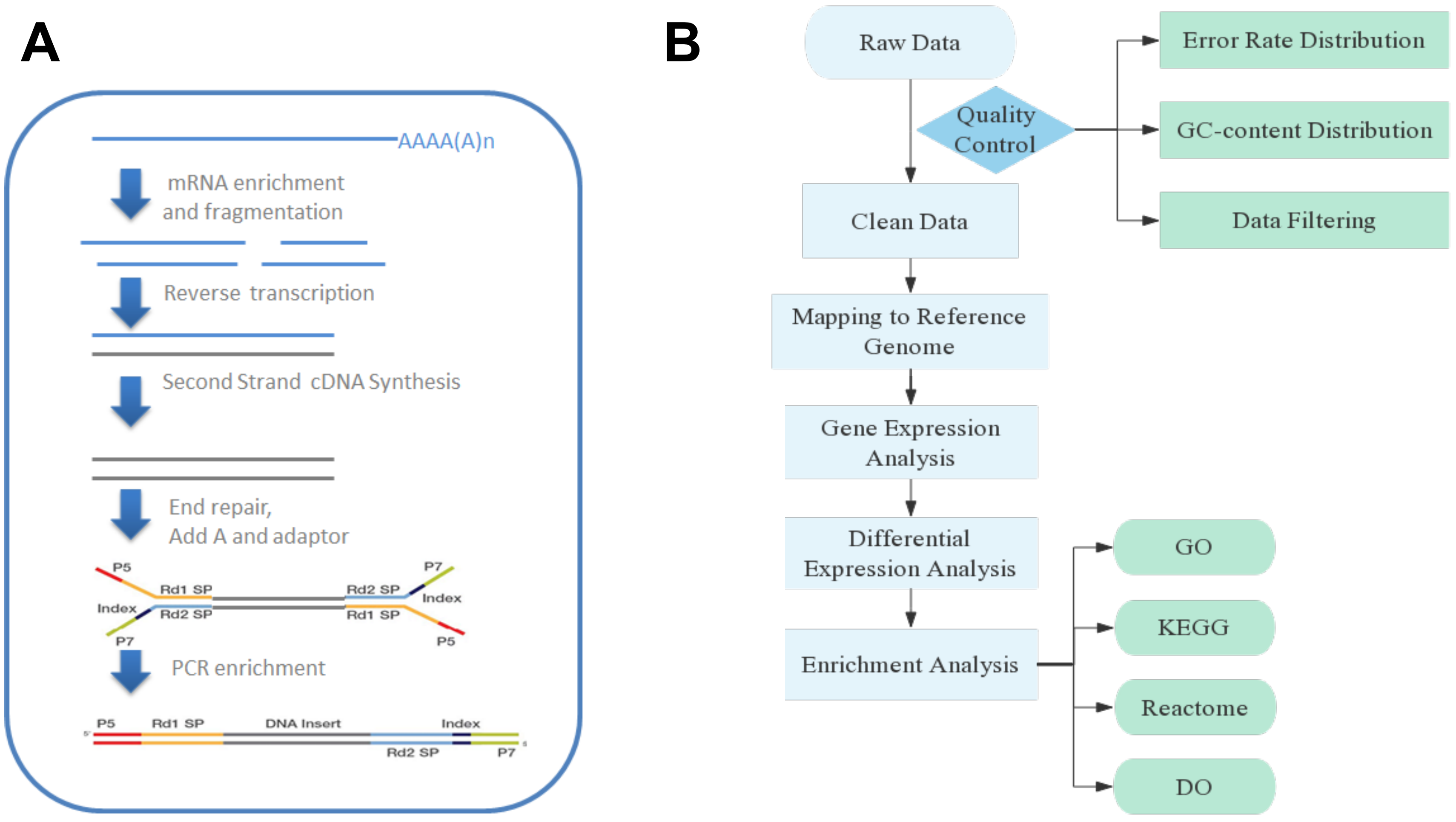

Figure S9. Generation of library and bioinformatic analysis. (A) Library construction. The cDNA is synthesized using random hexamer primers and MMLV. Second strand cDNA synthesis is performed using DNA polymerase I and RNAse H. The double-stranded cDNA is purified using AMPure XP beads and remaining overhangs converted into blunt ends. After adenylation of 3’ ends, NEBNext Adaptor with hairpin loop structure is ligated to prepare for hybridiation. Library fragments (preferentially 150-200bp) were purified with AMPure XP system (Beckman Coulter, Beverly USA). After PCR amplification fragments are purified using AMPure XP beads. The quality of the library was confirmed prior to sequencing (B) Bioinformatic analysis. Raw data underwent quality control and clean data was mapped to references and gene expression analysis performed. This illustration was generated by Novogene.

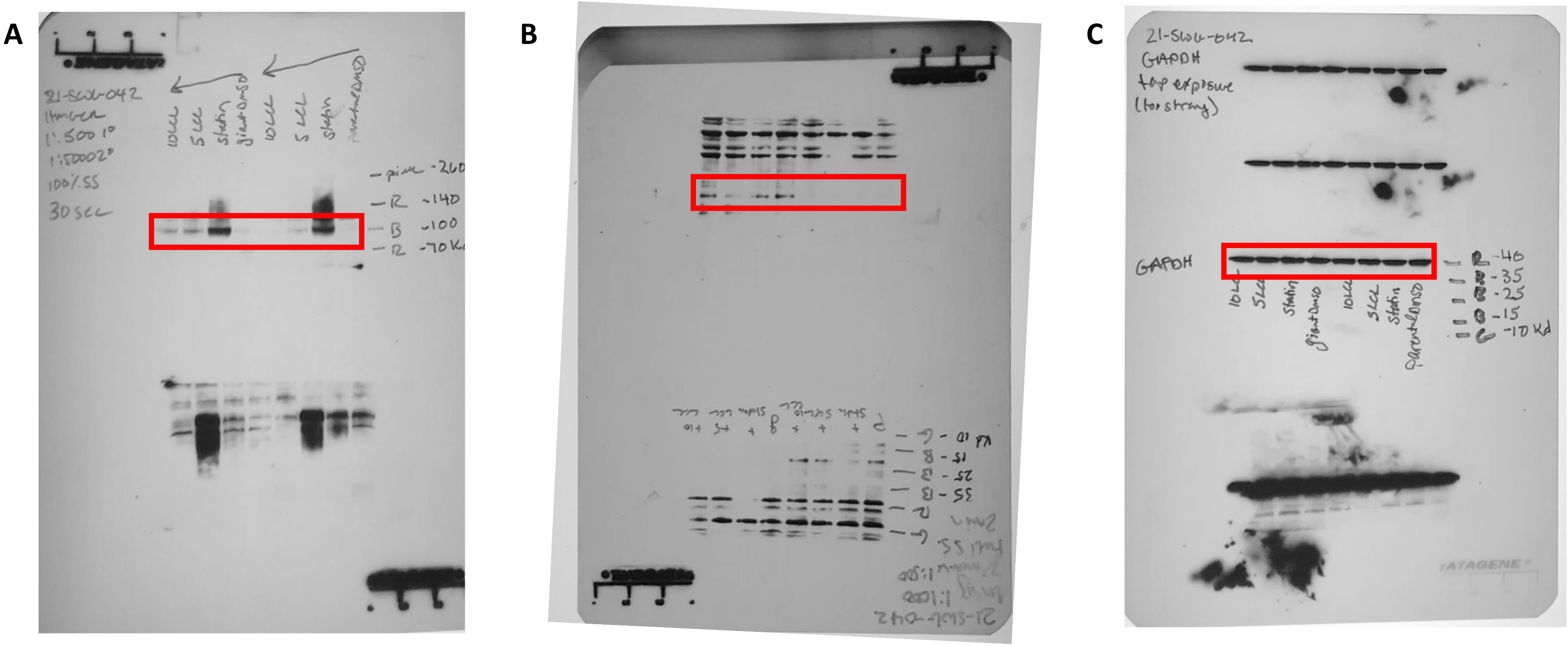

Figure S10. Uncropped Western blots for Figure 4A. (A) HMGCR ~95 kD flipped left to right to preserve legibility. Two exposures shown, one with markers. (B) INSIG1 ~30 kD with two exposures shown, one with markers. (C) GAPDH ~37 kD, multiple exposures, one with markers. All exposures done on film. Red boxes denote the exposures shown in Figure 4A.

A B

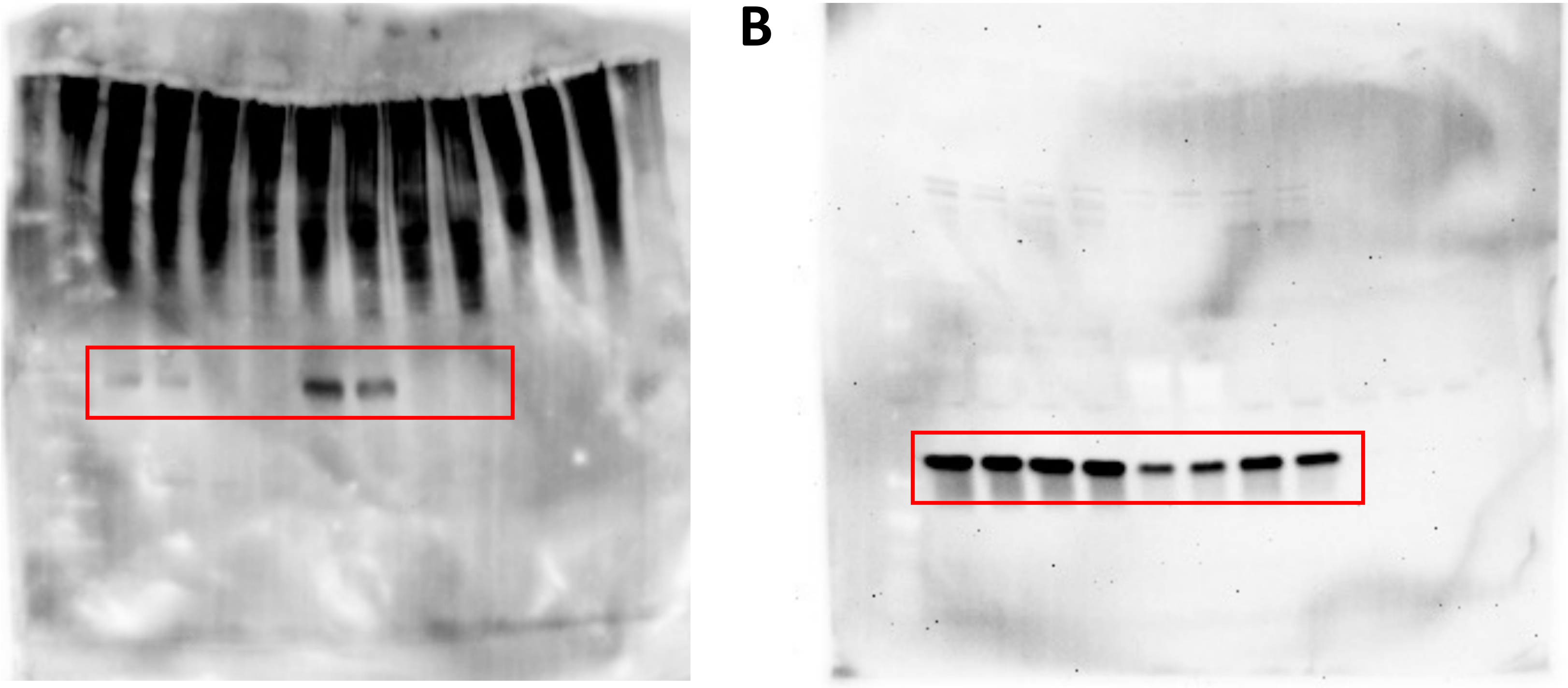

Figure S11. Uncropped Western blots for Figure 4E. (A) SR-B1 (B) GAPDH. Exposures performed on ChemiDoc.
